# Genomic Loci Modulating the Retinal Transcriptome in Wound Healing

**Published:** 2008-02-14

**Authors:** Félix R. Vázquez-Chona, Lu Lu, Robert W. Williams, Eldon E. Geisert

**Affiliations:** 1 Moran Eye Center, University of Utah, Salt Lake City, UT; 2 Key Laboratory of Nerve Regeneration, Nantong University, China; 3 Department of Ophthalmology, The Hamilton Eye Institute, University of Tennessee Health Science Center, Memphis, TN; 4 Center of Genomics and Bioinformatics, University of Tennessee Health Science Center, Memphis, TN; 5 Department of Anatomy and Neurobiology, University of Tennessee Health Science center, Memphis, TN

**Keywords:** retinal degeneration, CNS degeneration, genetic networks, QTL analysis, microarray

## Abstract

**Purpose:**

The present study predicts and tests genetic networks that modulate gene expression during the retinal wound-healing response.

**Methods:**

Upstream modulators and target genes were defined using meta-analysis and bioinformatic approaches. Quantitative trait loci (QTLs) for retinal acute phase genes (Vazquez-Chona et al. 2005) were defined using QTL analysis of CNS gene expression (Chesler et al. 2005). Candidate modulators were defined using computational analysis of gene and motif sequences. The effect of candidate genes on wound healing was tested using animal models of gene expression.

**Results:**

A network of early wound-healing genes is modulated by a locus on chromosome 12. The genetic background of the locus altered the wound-healing response of the retina. The C57BL/6 allele conferred enhanced expression of neuronal marker Thy1 and heat-shock-like crystallins, whereas the DBA/2J allele correlated with greater levels of the classic marker of retinal stress, glial fibrillary acidic protein (GFAP). *Id2* and *Lpin1* are candidate upstream modulators as they strongly correlated with the segregation of DBA/2J and C57BL/6 alleles, and their dosage levels correlated with the enhanced expression of survival genes (*Thy1* and *crystallin genes*).

**Conclusion:**

We defined a genetic network associated with the retinal acute injury response. Using genetic linkage analysis of natural transcript variation, we identified regulatory loci and can didate modulators that control transcript levels of acute phase genes. Our results support the convergence of gene expression profiling, QTL analysis, and bioinformatics as a rational approach to discover molecular pathways controlling retinal wound healing.

## Introduction

Transcriptome-wide analyses are defining the mechanisms that control retinal wound healing. Gene expression profiling of the injured retina revealed that changes in the transcriptome have spatial and temporal patterns (Yoshimura et al. 2003; Wilson et al. 2003; Vazquez-Chona et al. 2004; Chen et al. 2004; Ahmed et al. 2004; Gerhardinger et al. 2005; Rattner and Nathans, 2005). The spatial response to trauma involves changes that start at the stress site and then spread to include the entire retina (Vazquez-Chona et al. 2004). Transcriptome-wide changes are also highly regulated into three temporal patterns of expression—early acute (within hours), delayed subacute (within days), and late chronic phases (within weeks). Genes within each phase are functionally related and reflect known cellular changes. Transcriptome profiling of injured retina also revealed that global changes are highly similar across different injury models, including mechanical trauma, ischemia, and increased intraocular pressure (Vazquez-Chona et al. 2004, Yoshimura et al. 2003, Ahmed et al. 2004). Together the growing collection of retinal transcriptome profiles is cataloging the genes that underlie the biochemical and cellular changes of wound healing. The next level of analyses for expression data involves defining the networks and regulators that control specific wound healing processes such as cell death and tissue remodeling.

We used quantitative trait locus (QTL) analysis to discover the mechanisms regulating gene expression (Vazquez-Chona et al. 2005; Chesler et al. 2005). Expression QTL (eQTL) analysis combines transcriptome profiling with linkage analysis to reveal chromosomal loci modulating expression variability (Darvasi, 2003; Broman, 2005; Chesler et al. 2005). Expression genetics has been instrumental in identifying candidate genes underlying complex traits, including disease and behavior (Aitman et al. 1999; Karp et al. 2000; Berge et al. 2000). We previously found that a group of genes upregulated after central nervous system (CNS) injury (retina, brain, and spinal cord) also shares eQTLs in gene networks from the mouse forebrain (Vazquez-Chona et al. 2005). Our analysis relied on the BXD recombinant inbred (RI) strains derived from experimental crosses between the C57BL/6 and DBA/2J strains (Taylor et al. 1999; Peirce et al. 2004). Expression of acute phase genes in BXD RI mouse forebrains is modulated by eQTLs on chromosomes 6, 12, and 14 (Vazquez-Chona et al. 2005). This finding raised the hypothesis that these eQTLs also modulate the acute phase response of the retina.

In this study, we specifically tested the hypothesis that eQTLs on chromosomes 6, 12, and 14 modulate retinal wound healing. We determined the specificity of the loci by comparing eQTLs across functional groups and tissues. We defined candidate genes by using an integrated bioinformatic approach that combines online databases, single nucleotide polymorphism analysis, and gene expression profiling in the injured and developing CNS. We also tested the effect of expression dosage of candidate genes on wound healing. Our approach outlines an integrated bioinformatic and genetic approach that defines and tests networks of expression changes after retinal trauma.

## Results

### Specificity of expression loci

**Figure 1:**
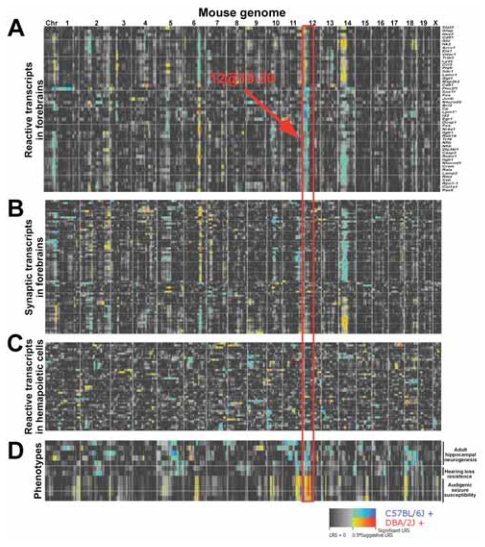
Chromosome 12 locus modulates expression of wound-healing genes. Quantitative trait locus (QTL) analysis maps the regulation of gene expression in BXD recombinant inbred (RI) strains. This regulation is based on the genetic correlation of expression (individual rows on the y-axis) to genomic markers across the mouse genome (x-axis). Blue hues represent correlations for elevated expression in mice with the C57BL/6 allele at a given locus, and orange hues represent correlations for elevated expression in mice with the DBA/2J allele. **A:** Expression of wound-healing genes in mouse forebrains is controlled by three eQTLs on chromosomes 6, 12, and 14. **B:** Synaptic-related genes in mouse forebrain are also controlled by eQTLs on chromosomes 6 and 14. **C:** Wound-healing genes shared no eQTLs in hematopoietic stem cells. **D:** Published data from phenotypes in BXD RI mouse strains further support that chromosome 12 locus also associates with neurological phenotypes. Affymetrix probe set identifiers and BXD Phenotype identifiers are listed in Supplementary Table 1. *Probe set for *Lpin1* is not available in Affy U34 chip, however, post meta-analysis predicted and experimental models of gene expression confirmed the role of *Lpin1* as a wound healing gene (see Figs. 4 and 5; and Supplementary Fig. 2).

We used QTL analysis to examine transcript modulation of CNS wound-healing genes (acute, subacute, and chronic phase genes). In forebrains of 35 BXD RI strains, a group of wound-healing genes (n = 44 genes; Supplementary Table 1) shared genetic linkages to eQTLs on chromosomes 6, 12, and 14 (Fig. 1A). To define the specificity of eQTLs, we compared loci controlling the expression of wound-healing genes to loci controlling synaptic-related genes. Wound-healing and synaptic-related genes shared eQTLs on chromosomes 6 and 14 (Fig. 1A and 1B). Chromosome 12 locus was specific to wound-healing genes. Specificity was further tested by comparing the loci controlling wound-healing genes in mouse forebrains and in hematopoietic stem cells; wound-healing genes shared no eQTLs in hematopoietic stem cells (Fig. 1C). Locus comparison across functional groups and across tissues revealed that the chromosome 12 locus specifically regulates the expression of a group of wound-healing genes.

**Figure 2:**
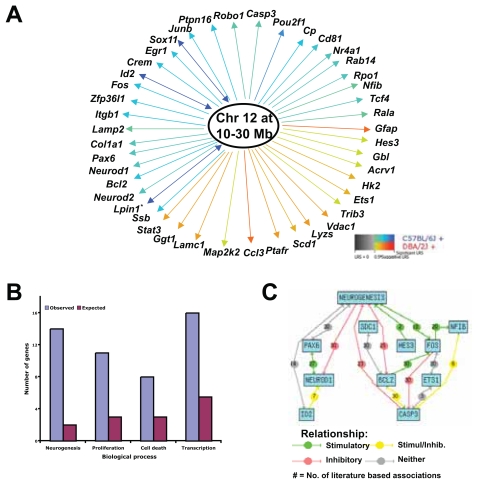
Chromosome 12 locus modulates transcription, differentiation, proliferation, and apoptotic mechanisms. **A:** Genetic networks were derived from transcripts sharing eQTLs as shown in Figure 1. Blue lines connecting specific genes to the locus represent correlations for elevated gene expression in mice with the C57BL/6 allele, and orange lines represent correlation for elevated gene expression in mice with the DBA/2J allele. Genes located within the eQTLs (cis-eQTLs) are indicated with a two-arrow line. **B:** The major functional themes described by the network’s gene functions are the regulation of transcription, differentiation, proliferation, and cell death. A nonbiased, statistical approach to defining the function of the network (n = 44 genes) is to compare the observed number of regulated genes as compared to the expected number in a population belonging to a particular functional category. For the chromosome 12 network, we observed 32% (14 out of 44 genes) of genes to be related to the regulation of neural development and differentiation. This percentage is higher than the percentage (7%) observed among the total population of retinal reactive transcripts and much higher than the percentage of expected genes in the entire genome. **C:** We queried the biological literature using text-mining tools to illustrate networks within the transcripts grouped into the neuro-genesis category (*Pax6, Neurod1, Neurod2, Id2, Nfib, Egr1, Hes3, Bcl2, Robo1, Ets1, Sox11, Casp3, Itgb1,* and *Sdc1*). The literature search documents the number of known molecular interactions of these genes, including activation and inhibition, that occur during neurogenesis. *Probe set for *Lpin1* is not available in Affy U34 chip, however, post meta-analysis predicted and experimental models of gene expression confirmed the role of *Lpin1* as a wound healing gene (see Figs. 4 and 5; and Supplementary Fig. 2).

The chromosome 12 locus modulates the expression of genes located outside the 10-to 30-Mb locus (*trans*-eQTL) and of genes within the locus (*cis*-eQTL). *Trans*-regulated genes included classic wound-healing genes: *Fos*, *Nr4a1*, and *Gfap. Cis*-regulated genes included nuclear genes *Id2*, *Lpin1*, and *Sox11* (Fig. 1A). Despite their diverse genomic locations, these transcripts were controlled by the segregating pattern of the C57BL/6 and DBA/2J alleles corresponding to chromosome 12 locus (Fig. 1A). On average, when the C57BL/6 alleles were present, *Fos*, *Nr4a1*, and *Id2* expressed higher levels than when the DBA/2J alleles were present. The converse was true for *Gfap*: when the DBA/2J alleles were present, *Gfap* expressed higher levels than when the C57BL/6 alleles were present. Since expression patterns of these transcripts correlated to the chromosome 12 locus, it follows that their expression patterns are correlated to each other: *Fos*, *Nr4a1*, and *Id2* were positively co-regulated by C57BL/6 alleles and were also positively correlated with each other (r > 0.68), whereas they were negatively correlated with *Gfap* (r < −0.52), a transcript positively correlated to the DBA/2J allele (Supplementary Table 2). Gene expression associations such as co-regulation by the same eQTL and significant expression correlation define genetic networks (Chesler et al. 2005). Therefore, wound-healing genes that were linked to chromosome 12 locus are part of a genetic network that controls their expression due to genetic differences between the parental strains (Fig. 2A). Since these transcripts were also differentially expressed in injured retina, this network may also control wound-healing events in the retina and elsewhere in the CNS.

### Biological processes controlled by chromosome 12 network

A simple approach to understanding the functional role of a genetic network is to examine the functions of gene products associated with the network. A nonbiased, statistical approach to defining the network’s function is to compare the observed and expected number of genes belonging to a particular functional category (Fig. 2B) (Zhang et al. 2005). For the network modulated by chromosome 12 locus, 32% of genes (14 out of 44) were related to the regulation of neural development and differentiation. This percentage was higher than the percentage of neural development genes in the mouse genome (5–10%). With this analysis, four functional themes emerged: regulation of transcription, cell death, cell proliferation, and neural development and differentiation. These functions are relevant to the early events of wound healing (Supplementary Fig. 1). The finding of significant functional themes within the network raised the possibility that these genes have known molecular associations. We queried the literature using text-mining tools to illustrate known biological interactions (Chen and Sharp, 2004). Within the group of genes related to neurogenesis, literature-based associations illustrated that transcription factor NeuroD1 activates pro-neural transcription factor PAX6 and that transcription repressor ID2 modulates NeuroD1. Additional associations were derived for transcripts involved in regulating transcription, cell cycle, and cell death (data not shown). This analysis suggests that text-mining approaches can support hypotheses and provide direction of potential molecular interactions. Together our data mining of biological concepts, gene function, and protein interactions suggests that the chromosome 12 network may regulate transcription, proliferation, apoptosis, and changes in phenotype (that is, de-differentiation) during retinal wound healing.

### Candidate genes

**Figure 3:**
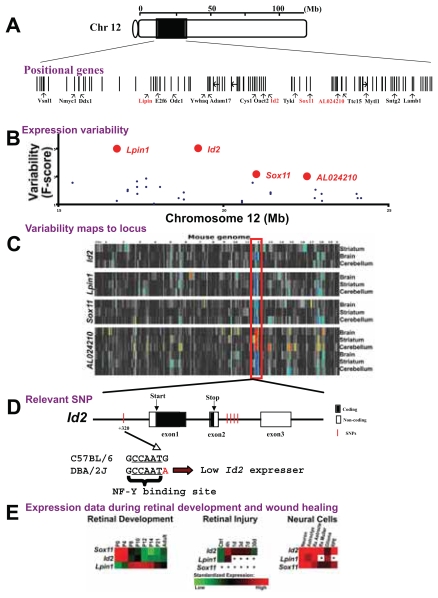
Candidate genes for chromosome 12 network. A candidate gene must have genetic polymorphisms that result in the expression variability of its own transcript (*cis*-eQTL). In addition, the candidate gene’s function must be consistent with molecular events that occur during retinal wound healing. **A:** Within the locus there are over 50 polymorphic genes. Within the enlarged interval, bars represent genes, and their spacing represents approximate location. Red bars represent genes that meet criteria. **B:** Transcript abundance variability in normal forebrain of BXD RI mouse strains is due to genetic polymorphisms between the parental C57BL/6 and DBA/2J mouse strains. The graph illustrates transcript abundance variability (y-axis) for genes (dots) within the 10- to 30-Mb interval of chromosome 12 (x-axis). *Lpin1* and *Id2* are polymorphic genes that displayed significant transcript variability and *cis*-eQTLs in normal forebrain of BXD mouse strains. **C:** We identified genes whose expression patterns in forebrain, cerebellum, and striatum of BXD mouse strains map to their gene locations. Within the interval, we identified in red the genes that display eQTLs in at least two brain regions. Linkage maps were generated using the Interval Mapping and Cluster Tree tools at GeneNetwork (GeneNetwork). **D:** The structure of the *Id2* gene illustrates an SNP at the promoter region and four SNPs within the second intron. The SNP within the promoter region (Ensembl SNPView ID rs4229289 and Celera SNP ID mC22302957) is located within a highly conserved region and is adjacent to a nuclear transcription factor Y (NF-Y) binding site (TRANSFAC ID M00185). In the diagram, filled and open boxes represent translated and untranslated regions **E:** We determined genes that are differentially expressed during retinal development and retinal healing. *AL024210* is highly homologous to human *MTCBP1* (NP_060739) and rat *Alp1* (NP_954528). Affymetrix probe set identifiers and BXD Phenotype identifiers are listed in Supplementary Table 1.

The next level of analysis defined the polymorphic gene(s) responsible for the eQTL. Within the chromosome 12 locus (10 to 30 Mb) there are over 50 polymorphic genes (Fig. 3A). We focused on those polymorphic genes whose expression patterns in the CNS of BXD mouse strains were variable and mapped within the eQTL (Fig. 3B and 3C). In forebrain, the expression variability of genes *Lpin1*, *Id2*, *Sox11*, and *AL024210* mapped within the locus. *Lpin1*, *Sox11*, *AL024210*, and *Id2* had their gene location on chromosome 12 at 15.9, 23.9, 25.2, and 21.6, respectively (Fig. 3B). Their DNA sequences between the C57BL/6 and DBA/2J genomes display single-nucleotide polymorphisms (SNPs) in their coding and regulatory regions (SNPBrowser, accessed November 2005, SNPView, v33). For example, *Id2* had SNPs on its promoter (Celera SNP ID mC22302957), introns (Celera SNP IDs mCV22302969, 77–79, 89), and third exon (mCV22302990, 2991, 3002, 3003) (Fig. 3D). Genetic variability can explain the expression variability and strong linkage of *Lpin1*, *Sox11*, *AL024210*, and *Id2* to their gene locations (*cis*-eQTLs). These genes also displayed *cis*-eQTLs across brain tissues including forebrain, cerebellum, and striatum (Fig. 3C). The presence of polymorphisms and *cis*-eQTLs across CNS tissues suggested that *Lpin1*, *Sox11*, *AL024210*, and *Id2* were strong candidate modulators of the chromosome 12 network.

Bioinformatic approaches helped us determine whether polymorphisms can alter functional motifs. For example, the SNP on the *Id2* promoter (Celera SNP ID mC22302957) was adjacent to a putative nuclear factor Y (NF-Y) transcription factor binding site (Fig. 3D; (Vazquez-Chona et al. 2005)). *Lpin1* had exonic SNPs near the 5′splice site of intron 11 (Celera SNP ID mCV22346966) and near splice sites on exon 1 (Celera SNP IDs mCV22347703 and mCV22347384). Genetic variants in a transcription factor-binding site and at splice locations can lead to differences in mRNA levels. These differences made the genetic mapping possible. Using bioinformatic tools, we determined that the SNPs on the candidate genes may affect functional domains such as transcription binding sites and splice sites. These observations predict a potential mechanism by which SNPs interfere with the expression of candidate genes.

The role of a gene as a modulator of the chromosome 12 network, and potentially of retinal wound healing, was bolstered by determining if its gene product played a role in CNS development and disease. Using publicly available data on mouse retinal development (Dorrell et al. 2004), we determined that *Lpin1*, *Id2*, and *Sox11* were expressed by the developing retina and had well defined patterns of expression (Fig. 3E; note that *AL024210* levels were below detectable thresholds). Using our microarray data set (Vazquez-Chona et al. 2004) and reverse transcriptase polymerase chain reaction (RT-PCR) data on rat retinal injury, we found that *Lpin1* and *Id2* were acute phase genes (Fig. 3E; the *Sox11* U34 probe set displayed signals below noise thresholds, and there was no probe set for *AL024210*). *Lpin1*, *Id2*, and *Sox11* were expressed in reactive glia of optic nerve heads (Yang et al. 2004) and diabetic retinas (Gerhardinger et al. 2005). Our meta-analyses of publicly available data revealed that *Lpin1*, *Id2*, and *Sox11* were differentially expressed during retinal development and trauma. Together these results documented that *Lpin1*, *Id2*, and *Sox11* are candidate genes, because they displayed (1) *cis*-eQTLs in the brain, cerebellum, and striatum; (2) polymorphisms between parental strains; (3) differential expression during retinal development and retinal healing; and (4) moderate to high levels of expression in normal and reactive neural cells. However, we cannot exclude the role of predicted gene *AL024210* or an unknown gene within the locus as a potential regulator of chromosome 12 network.

### Testing candidate genes

**Figure 4:**
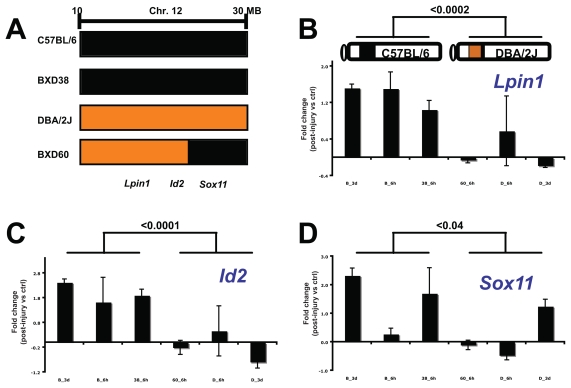
Expression of *Id2* and *Lpin1* correlates with the segregation of chromosome 12 locus. To test the hypothesis that *Id2, Lpin1,* or *Sox11* modulates the eQTL, we used the natural range of expression in BXD RI strains. **A:** We compared the wound-healing response of retinas in strains with the C57BL/6 allele (C57BL/6 and BXD38) to strains with the DBA/2J allele (DBA/2J and BXD60). The BXD60 strain has an additional recombination between genetic markers at 22 and 30 Mb. **B, C, D:** Acute and subacute expression changes for *Lpin1, Id2,* and *Sox11* were measured in these strains. Fold changes represent expression differences between normal and injured conditions (log_2_ scale). Averages are expressed as the mean ± SEM. Significance was measured using Student’s t-test.

We tested the hypothesis that *Id2*, *Lpin1*, or *Sox11* modulates the eQTL on chromosome 12 by using an animal model of expression dosage. In BXD RI strains, the segregating pattern of C57BL/6 and DBA/2J alleles generates a natural range of transcript and protein expression (Supplementary Fig. 2A and 2B). We used four BXD strains: parental strains C57BL/6 and DBA/2J; BXD38 strain with the C57BL/6 allele; and BXD60 strain with an additional recombination between genetic markers at 22 and 30 Mb (the proximal and distal regions being DBA/2J and C57BL/6, respectively) (Fig. 4A). Our goal here was to determine if the expression of candidate genes after injury correlated with the segregation of alleles.

Quantitative RT-PCR analyses showed that genetic background altered the expression change of candidate genes during retinal wound healing. We measured transcript levels in the acute phase (6 h) and subacute phase (3 d) that occur following optic nerve crush (Fig. 4). For *Id2* and *Lpin1*, C57BL/6 and BXD38 retinas displayed greater fold changes than did DBA/2J and BXD60 retinas (p < 0.02) (Fig. 4B and 4C). *Lpin1* in C57BL/6 retinas showed a subacute upregulation of 1.53 ± 0.14 (p < 0.001), and *Lpin1* in DBA/2J retinas showed a subacute down-regulation of 5.56 ± 2.39 (p < 0.05). In models of retinal tear and toxic injury, higher *Id2* and *Lpin1* expression levels and fold changes were also observed in BXD38 retinas relative to DBA/2J retinas (Supplementary Fig. 2C and 2D). These results suggested that *Id2* and *Lpin1* had larger fold changes which were associated with the C57BL/6 allele (C57BL/6 and BXD38 strains) and not with the DBA/2J allele. The additional recombination between genetic markers at 22 and 30 Mb in the BXD60 strain further showed that lower *Id2/Lpin1* fold changes correlated with the DBA/2J genetic background at the proximal region (*Id2* and *Lpin1* are located at 15.9 and 21.6 Mb). *Sox11* expression in injured BXD retinas did not correlate with the segregating patterns of alleles. For example, in both the C57BL/6 and DBA/2J retinas, *Sox11* displayed significant upregulation during the subacute phase (2.33 ± 0.43 and 1.35 ± 0.20; p < 0.05). Based on expression data from injured BXD retinas, the expression patterns for *Id2* and *Lpin1* correlated positively with the C57BL/6 allele. These results suggest that *Id2* and *Lpin1* are our best current candidate genes.

### Chromosome 12 network modulates retinal wound healing

**Figure 5:**
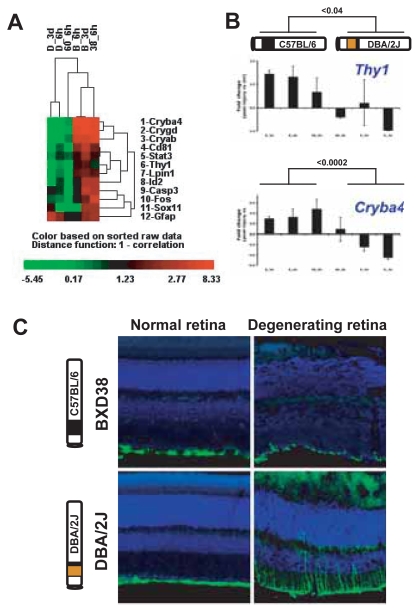
Higher levels of *Id2* and *Lpin1* (C57BL/6 allele) correlated with higher levels of survival markers. To investigate the role of chromosome 12 locus during retinal injury, we examined the wound-healing response of retinas that expressed high levels of *Id2/Lpin1* (strains with the C57BL/6 allele: C57BL/6 and BXD38) and compared this to low levels of *Id2/Lpin1* (strains with the DBA/2J allele: DBA/2J and BXD60). Acute-phase and subacute-phase mRNA levels were measured 6 h and 3 d after an optic nerve crush. **A:** Clustered dendogram shows the correlation across experiments (columns) and across genes (rows) in terms of distance (1 — correlation). **B:** Retinas with the C57BL/6 allele expressed higher fold changes of *Thy1* and *Cryab* than did retinas with the DBA/2J allele. Fold changes represent expression differences between normal and injured conditions (log_2_ scale). Averages are expressed as the mean ± SEM. Significance was measured using Student’s t-test. **C:** Genetic-based differences in GFAP immunoreactivity. Six days after an optic nerve crush, we measured stress levels using the classic stress marker GFAP, a cytoskeletal protein normally expressed by astrocytes and the end-feet of Muller cells.

To investigate the role of chromosome 12 locus, we compared the wound-healing response of retinas that expresses high levels of *Id2*/*Lpin1* (strains with the C57BL/6 allele) to retinas expressing low levels of *Id2*/*Lpin1* (strains with the DBA/2J allele) (Fig. 5). Using cluster analysis, we compared patterns in fold changes for markers of acute phase (*Fos* and *Stat3*), apoptosis (*Casp3*), gliosis (*Gfap* and *Cd81*), and survival (*Thy1* and *Cryab)*. We also measured expression levels for members of the crystallin family (*Cryab*, *Cryba4*, and *Crygd*), because they represent a significant component of the retinal wound-healing response (Vazquez-Chona et al. 2004) and are important in the rescue of the degenerating retina (Otani et al. 2004). Expression patterns in each experiment (n = 6) were more influenced by the genetic background than by the wound-healing phase (Fig. 5A). The correlation between acute phase expression between the C57BL/6 and DBA/2J strains was strongly negative (r = −0.87). In contrast, the correlation within strain experiments was strongly positive. For the C57BL/6 strain, the correlation between acute phase and subacute phase expression values was 0.96. Similar correlations were observed in the DBA/2J strain (r = 0.92). In general, experiments with the same allele clustered together. These results suggested that the genetic background of chromosome 12 locus influences the expression of wound-healing genes.

The genetic background of chromosome 12 locus significantly altered the expression of genes related to survival (Fig. 5A). Higher *Id2* and *Lpin1* fold changes strongly correlated with higher fold changes in ganglion cell gene *Thy1* (r = 0.86 and r = 0.94, respectively) and crystallin genes *Cryab*, *Cryba4*, and *Crygd* (0.82 > r < 0.89). Retinas with the C57BL/6 allele also expressed higher fold changes of *Thy1* and crystallins than did retinas with the DBA/2J allele (p > 0.03 and p < 0.04, respectively; Fig. 5B). In contrast, the apoptotic gene *Casp3* had a weak correlation with *Thy1* (r = 0.65) and had no significant difference in fold changes due to genetic background. Based on these results, the C57BL/6 allele (greater *Id2*/*Lpin1* fold changes) correlated with higher expression levels of survival genes including the ganglion cell gene *Thy1* and crystallin genes (*Cryab*, *Cryba4*, and *Crygd*).

The chromosome 12 locus also had differential effects on the activation of retinal glial cells. We examined fold changes for classic markers of glial activation: STAT3, CD81, and GFAP. *Stat3* expression patterns strongly correlated with those of *Id2* and *Lpin1* (r = 0.87 and r = 0.88). This finding was significant since STAT3 signaling is important for the functional recovery of the CNS (Okada et al. 2006). *Cd81* expression patterns also strongly correlated with those of *Id2* and *Lpin1* (r = 0.9215 and r = 0.9483). This finding was significant since CD81 antigen is involved in the negative regulation of glial proliferation (Geisert et al. 1996). In contrast, *Gfap* expression displayed a weak, but negative, correlation with *Id2* and *Lpin1* (r = −0.23 and r = −0.29). To further explore the relationship between chromosome 12 regulation and GFAP expression, we compared GFAP immunoreactivity across genetic backgrounds. GFAP is a cytoskeletal protein normally expressed by astrocytes and the end-feet of Muller cells. Six days after an optic nerve crush, the central and the peripheral retina of the low *Id2*/*Lpin1* expresser (DBA/2J strain) displayed increased GFAP immunoreactivity: Muller cells increased the expression of GFAP, as seen by the increased immunoreactivity along the radial branches of Muller cells (Fig. 5C, two animals per strain). In contrast, the high *Id2*/*Lpin1* expresser (the BXD38 strain) at 6 days after injury displayed GFAP labeling of Muller cells only at the peripheral retina and a delayed upregulation of GFAP in the central retina. Immunoreactivity and mRNA results suggested that the C57BL/6 allele (high *Id2*/*Lpin1* expresser) displayed smaller changes in GFAP expression than did the DBA/2J allele. Moreover, the C57BL/6 allele (high *Id2*/*Lpin1* expresser) displayed greater changes in glial cells for the protective STAT3 signaling and for the negative regulator of glial proliferation CD81.

## Discussion

Here we predicted and tested a genetic network that modulates the expression of genes involved in the early CNS wound-healing response. Our approach to defining networks controlling retinal wound healing involved (1) highlighting networks of highly co-expressed genes in different models of injury and different CNS tissues, (2) defining the location of upstream regulators using QTL analysis, (3) predicting upstream modulators using bioinformatic tools, and (4) testing the effect of candidate modulators on wound healing by using animal models of gene dosage. Using this approach, we found that a network of early wound-healing genes is modulated by an eQTL on chromosome 12, 10–30 Mb away from the centromere. The genetic background of the eQTL altered the wound-healing response of the retina. The C57BL/6 allele conferred enhanced expression of neuronal marker *Thy1* and heat-shock-like *crystallins*, whereas the DBA/2J allele correlated with greater levels of the classic marker of retinal stress, glial fibrillary acidic protein (GFAP). *Id2* and *Lpin1* are our candidate upstream modulators as they strongly correlated with the segregation of DBA/2J and C57BL/6 alleles, and their dosage levels correlated with the enhanced expression of survival genes (*Thy1* and *crystallin genes*). Our results support the convergence of gene expression profiling, QTL analysis, and bioinformatics as a rational approach to discover molecular pathways controlling retinal wound healing.

Previous studies identified three eQTLs that modulate the expression of wound healing genes (Vazquez-Chona et al. 2005). Here we showed that the chromosome 12 network modulates genes involved in the transcription, differentiation, proliferation, and apoptosis of neural cells. In the injured retina, these genes are differentially expressed during the early acute and delayed subacute phases (Supplementary Fig. 1). The function and temporal expression of the network’s genes are consistent with early wound-healing events, mainly neuronal cell death and glial activation. Published data also support the hypothesis that chromosome 12 network modulates the CNS response to trauma. In BXD RI mouse strains, the DBA/2J allele for the chromosome 12 locus is one of the loci associated with susceptibility to noise-induced cell death of spiral ganglion cells, the cells innervating auditory receptor cells (Fig. 1D) (Willott and Erway, 1998). The DBA/2J allele also predisposes susceptibility to generalized convulsions when exposed to intense auditory stimulation (Neumann and Collins, 1991). In contrast, the C57BL/6 allele is one of the loci that confers enhanced neurogenesis and survival of new neurons and astrocytes in adult hippocampus (Kempermann and Gage, 2002). Similarly, the C57BL/6 background confers resistance to glutamate-induced neurotoxicity in brain and spinal cord (Schauwecker and Steward, 1997; Inman et al. 2002). Together these data suggest that the chromosome 12 network displays a prominent role in CNS wound healing.

Chromosome 12 network represents a novel mechanism that may explain differences in the response to retinal injury in murine models. Specifically, our data showed that the DBA/2J allele confers susceptibility to neurodegeneration and glial reactivity induced by different experimental models of retinal injury, including toxic injuries and optic nerve crush. The DBA/2J background also displays susceptibility to retinal ganglion cell death due to elevated intraocular pressure, a hallmark of glaucoma (John, 2005). Understanding the genetic factors that control neuronal degeneration and glial reactivity in BXD RI mice can help elucidate the mechanisms that control susceptibility to glaucoma in humans. Our results suggest that the C57BL/6 background may confer resistance to neurodegeneration by enhancing the expression of survival factors such as acute-phase factor STAT3 and crystallins. Several experiments demonstrated the survival function of STAT3 and crystallins. Genetic inactivation experiments in mice showed that STAT3 is important for the functional recovery of CNS (Okada et al. 2006). The rescue of degenerating retinas by stem cell therapy upregulates crystallins (Otani et al. 2004). Ganglion cell and photoreceptors can upregulate crystallin expression after retinal injury (Vazquez-Chona et al. 2004, Yoshimura et al. 2003, Sakaguchi et al. 2003). Thus the enhanced expression of survival markers in the C57BL/6 allele relative to the DBA/2J allele suggests that the chromosome 12 locus may represent the location for genetic variants responsible for susceptibility to some forms of neurodegeneration. This chromosomal interval has a high degree of homology (that is, synteny) with intervals on human chromosome 2 (1 to 18 Mb or p25 to p24.3) and on rat chromosome 6 (34 to 48 Mb or q14 to q16) (MapViewer, accessed January 2006). In these syntenic regions, the gene order and gene homology are highly conserved. As we enhance the resolution of our linkage mapping by increasing the number of unique BXD RI strains (Peirce et al. 2004), it is in principle possible to shorten QTL intervals to the size of small numbers of genes and perhaps relate transcript variability down to a single SNP.

Using an integrated bioinformatic approach, we examined each polymorphic gene within the locus for *cis*-eQTLs, biological relevant SNPs, and biological significance to CNS wound healing. These analyses suggested that *Id2* and *Lpin1* were the best candidate genes. Their expression levels in forebrain, cerebellum, and striatum of BXD RI mouse strains have strong linkages to their locus (that is, *cis*-eQTLs). This means that a genetic variant (or variants) within or near the loci of *Id2* and *Lpin1* affects their own expression. Our computational analyses showed that SNPs in *Id2* and *Lpin1* may affect DNA binding sites and splice variants, respectively. Preliminary in vivo data suggested that *Id2* and *Lpin1* were candidate upstream modulators of the chromosome 12 network and modulators of retinal wound healing. In normal and injured retinas, *Id2* and *Lpin1* were more highly expressed in strains carrying the C57BL6 allele than in strains carrying the DBA/2J allele. Higher levels of *Id2* and *Lpin1* correlated with higher levels of neuronal marker Thy1 and heat-shock-like crystallin genes (*Cryab*, *Cryba4*, and *Crygd*). Our bioinformatic-based data and preliminary in vivo data provided the rationale for focusing on transcription regulator *Id2* and the nuclear protein *Lpin1* as the best, current candidate genes.

The known functions of ID2 are consistent with the potential roles of chromosome 12 during the early CNS response to injury: regulation of transcription, differentiation, proliferation, and apoptosis. The mining of literature and publicly available microarrays revealed that *Id2* is expressed by reactive glia in brain, spinal cord, and optic nerve head (Tzeng et al. 2001; Tzeng et al. 1999; Aronica et al. 2001; Hernandez et al. 2002). *Id2* is also upregulated as an immediate early gene in injured retina, brain, and spinal cord (Vazquez-Chona et al. 2005). Moreover, *Id2* silencing in cultured cerebellar astrocytes resulted in a mitigated reactive response, mainly a decrease in migration and proliferation (see Supplementary Fig. 3) (Vazquez-Chona, 2006). In its classical function, ID2 modulates neural and glial differentiation via the negative regulation of basic helix-loop-helix (bHLH) transcription factors (for example, NeuroD and Olig) (Ross et al. 2003, Liu and Harland, 2003; Wang et al. 2001). ID2 binds to bHLH transcription factors to inhibit their binding to E-box sequences, defined by the CANNTG motif (Yokota and Mori, 2002). E-boxes are present in promoters of many pro-neural differentiation genes (for example, *Pax6*) (Ross et al. 2003). These data suggest that ID2 can modulate activation of retinal cells by regulating E-box mediated transcription. Several of the network genes have E-boxes in their promoters including *Fos* (Chaudhary and Skinner, 1999), *Gfap* (Tzeng, 2003), *Sdc1* (Vihinen et al. 1996), *Pax6* (Marsich et al. 2003), and *Cp* (Vazquez-Chona, 2006). For example, ID2 modulates the gene expression of *Fos* during Sertoli cell differentiation (Chaudhary and Skinner, 1999). ID2 can also promote proliferation by inhibiting the E-box mediated transcription of cell cycle inhibitors (for example, p21CIP1/WAF1, p15INK4B, and p16INK4B) (Pagliuca et al. 2000). Alternatively, ID2 may stimulate proliferation by binding to the unphosphorylated Rb protein, allowing the release of the transcription factor E2F. E2F in turn activates genes involved in G1-S phase transition and hence proliferation (Yokota and Mori, 2002). In our studies, *Id2* silencing in cultured cerebellar astrocytes decreased the number of cells entering the S phase (see Supplementary Fig. 3) (Vazquez-Chona, 2006). ID2 can also negatively modulate apoptosis. In animals with *Id2* null mutation, Sertoli cells and mammary epithelial cells display increased levels of apoptosis relative to wild-type animals (Yokota and Mori, 2002). The increased apoptosis in *Id2*−/− animals suggests that ID2 may be required as a survival factor for some cell types (Yokota and Mori, 2002). In our studies, higher levels of *Id2* correlated with higher levels of survival gene *Crygd* and lower levels of acute-phase genes *Fos* and *Stat3*, apoptotic gene *Casp3*, and gliosis associated genes *Gfap* and *Cd81*. Together the functions of ID2 and these data suggest that ID2 is an ideal candidate upstream modulator of the chromosome 12 network.

The role of lipin 1 during retinal wound healing is not clear. Lipin 1 belongs to a novel family of nuclear proteins that are involved in adipose tissue development and insulin resistance. Lipin proteins share three conserved domains: a lipin, N-terminal conserved region; a nuclear localization sequence; and an LNS (Lipin/Ned1/SMP2) conserved region. Mutations in the *Lpin1* gene lead to fatty liver dystrophy (fld) in *fld* mice, characterized by loss of body fat, fatty liver, hypertriglyceridemia, and insulin resistance (Peterfy et al. 2001). *Lpin1* null mutations also lead to peripheral neuropathy due to the dysregulation of a battery of genes required for the regulation of storage lipid metabolism in both the endoneurium and peri/epineurium (Verheijen et al. 2003). The mechanisms of action by lipin proteins are unknown. A study of yeast lipin protein, Ned1, revealed that lipin proteins can associate with factors involved in nuclear transport and chromosome segregation (Tange et al. 2002). Gene expression patterns in the developing retina suggest that *Lpin1* is expressed during the time of Muller cell birth and differentiation (P8-P14) (Retina Developmental Gene Expression, probe set 98892_at (RetDevExpression, accessed January 2005); Mouse Retina SAGE Library, Mm.153625 (RetSAGE, Accessed October 2005)). We will use in situ hybridization to determine the cellular source of *Lpin1* mRNA. We will also use *fld* mice to determine the role of Lipin 1 during wound healing.

Our work makes two clear contributions to the field of CNS wound healing research. At one level, our work describes an integrated approach of gene expression profiling and higher-level bioinformatic analyses to define and test networks. This approach is valuable because it suggests how researchers might develop a package of capabilities to enable a systematic reconstruction of pathways related to their field. Our second contribution is finding a locus that modulates the early wound-healing response of the retina. Computational analyses and molecular manipulation of this locus will help define the regulatory gene(s) modulating the response of mammalian CNS to trauma and chronic stress.

## Methods

### Online bioinformatic resources

This study was designed to define regulatory mechanisms from our microarray data of injured retina (Vazquez-Chona et al. 2004). This data set surveyed the temporal gene expression profiles of rat retinal wound healing after a mechanical tear (4 h and 1, 3, 7, and 30 days). Our main strategy to discover upstream regulators of injury genes involved (1) defining eQTLs that modulate injury gene expression, and (2) defining candidate genes for each eQTL by using publicly available databases and computational tools. We combined a suite of bioinformatic tools (Table 1) previously described by our work (Vazquez-Chona et al. 2005; Chesler et al. 2005).

### Meta-analyses of microarray data sets

For publicly available microarray data, we obtained the raw data (CEL files), determined signal values by using Microarray Suite 5.0 (MAS 5.0; Affymetrix, Santa Clara, CA), transformed signals to a log scale (base 2), and normalized microarray mean intensity to 8 as described previously (Vazquez-Chona et al. 2004). These transformations yielded signal intensities ranging from 1 to 18 relative units of fluorescence. Further analyses included only transcripts with medium to high abundance (that is, signals greater than 8.64 (Vazquez-Chona et al. 2004)). A gene was considered expressed in a tissue if it displayed medium to high abundance. Wound-healing genes were defined by comparing injured retinal time points versus normal retina and using three criteria: fold changes >*|±2|*, Student’s t-test (P < 0.05), and genes with moderate to high expression levels. Comparison of rat genes across species was assayed by finding the corresponding ortholog’s probe set(s) by using Affymetrix NetAffx Analysis Center (NetAffx, Accessed June 2005) and Ensembl AffyProbe (v33) database. Since gene names may differ across species and multiple probe sets may be available, we included the probe set identifier when necessary. Gene expression was clustered with CLUSFAVOR by using principal component analysis (PCA) (Peterson, 2002).

### QTL analyses

Regulatory loci were defined using GeneNetwork (GeneNetwork), which is maintained by members of our group (RWW and LL). GeneNetwork is a suite of databases and analysis software that identifies loci that control transcriptome differences in brain regions in mouse strains derived from C57BL/6 (B) and DBA/2J (D) mice. Details on the methods, data, and analyses are available at GeneNetwork (GeneNetwork, Chesler et al. 2005; Bystrykh et al. 2005). We used the published expression genetics database for mouse forebrain (UTHSC Brain mRNA U74Av2 HWT1PM, December 2003) (Chesler et al. 2005), which consists of parental strains, the F1 hybrid, and 32 BXD RI strains (a total of 35 isogenic lines) (Chesler et al. 2005). Using linkage analysis and a genetic map consisting of 779 fully genotyped markers, GeneNetwork correlates expression variability to genotypes at locations throughout the mouse genome. The average distance between adjacent markers is approximately 4 Mb. Variability across strains was measured using analysis of variance (ANOVA) testing the between-strain variance compared with the total variance for 100 arrays from 35 mouse strains. The degrees of freedom for the between-group and total variance were 34 and 99. Strain-specific variation was significant (p < 0.05) when F_34,99_ > 1.5. We also supplemented the forebrain data with the published database for mouse hematopoietic stem cells (GNF Hematopoietic Cells U74Av2 RMA, March 2004) (Bystrykh et al. 2005), as well as the unpublished databases for mouse striatum (HBP/Rosen Striatum M430V2 RMA, April 2005) and mouse cerebellum (SJUT Cerebellum mRNA M430 RMA, March 2005). We also related gene expression phenotypes to mouse neurological phenotypes by using the BXD Published Phenotypes Database (BXDphenotypes, Accessed November 2005).

### Computational analysis of gene sequence, function, and expression

Gene location and structure were determined using Genome Browser (GenomeBrowser, Mouse May 2004 assembly) and Ensembl Genome Browser (Ensembl, v33). Transcription factor binding sites were defined using the TRANSFAC 5.0 database available through the MOTIF website (MOTIF, Accessed November 2005). Highly conserved regions were defined using the Conservation tool in USCS Genome Browser (GenomeBrowser, Mouse May 2004 assembly). Gene expression patterns in the developing mouse retina were obtained using Retina Developmental Gene Expression (RetDev-Expression, accessed January 2005). Gene expression patterns in the injured rat retina were obtained using our data available online at Gene Expression Omnibus (GEO, GSE1001). Gene expression levels from cultured mouse neurons (Kraft et al. 2004), mouse astrocytes (Kraft et al. 2004), rat Muller cells (Gerhardinger et al. 2005), human optic nerve astrocytes (Yang et al. 2004), human U87 glioma cells (unpublished data, Eldon Geisert), and rat RPE cells (unpublished data, Eldon Geisert) were obtained from published and unpublished data. To determine functional themes within a network of genes, we retrieved the Gene Ontology annotation data using WEB-based GEne SeT AnaLysis Toolkit (WebGestalt, Accessed October 2005). To mine literature abstracts, we used the online tool Chilibot (Chilibot, Accessed October 2005).

### Animals, anesthesia, and surgery

Six strains of mice were used for this study (n = 72 mice). These included the DBA/2J strain (n = 20), C57BL/6 strain (n = 20), BXD38 strain (n = 20), BXD60 strain (n = 6), and F1 progeny from a DBA/2J and C57BL/6 cross (n = 6). Strains were purchased from The Jackson Laboratory (Bar Harbor, ME), and colonies were maintained at the University of Tennessee Health Science Center. BXD38 and BXD60 strains are inbred lines derived from brother-sister matings starting from F2 intercrosses (Taylor et al. 1999, Peirce et al. 2004). For all experiments, we used adult male mice (7–10 weeks old, 22–25 g). All protocols used in this study were approved by the Animal Care and Use Committee of the University of Tennessee Health Science Center and were in accordance with the Institute for Laboratory Animal Research and with the ARVO Statement for the Use of Animals in Ophthalmic and Vision Research. Prior to any retinal injuries, mice were deeply anesthetized with avertin (1.25% 2,2,2-tribromoethanol and 0.8% tert-pentyl alcohol in water; 0.8–1.0 ml, intraperitoneal injection). Injured animals and noninjured animals used for gene and protein expression were deeply anesthetized with CO_2_. Animals used for immunohistochemistry were deeply anesthetized with avertin (1.0–1.2 ml, intraperitoneal injection) prior to perfusion. Retinal wound healing was induced via retinal tears, toxic injuries, and optic nerve crushes. Tears were induced by penetrating the pars plana with a 27-gauge needle and scraping the superior nasal retina as previously described (Vazquez-Chona et al. 2004). Toxic injuries were induced by injecting kanaic acid into the vitreous (2 ng diluted in sterilized saline). Optic nerve crushes were performed using a binocular operating microscope, incising the conjunctiva of eye, exposing the optic nerve, and pressing on the nerve with a cross-action forceps for 15 seconds.

### Real time RT-PCR

Total RNA from mouse retina was isolated with TRIzol (Life Technologies, Carlsbad, CA), treated with RQ1 RNase-Free DNase (Promega, Madison, WI), and queried with the Agilent 2100 bioanalyzer (Agilent Technologies, Palo Alto, CA). Transcript levels were measured using real-time quantitative reverse transcriptase polymerase chain reaction (qRT-PCR). We performed primer design, qRT-PCR reaction, and RT-PCR analysis as described in Vazquez et al. 2004. Nucleotide sequences are shown in Supplementary Table 3. We performed qRT-PCR reactions in the iCycler (BioRad, Richmond, CA) by using the reagents in the SYBR Green RT-PCR kit (Applied BioSystems, Warrington, UK). To determine the relative change in gene expression, we compared the number of cycles needed to reach the midpoint of the linear phase using the iCycler analysis software. All observations were normalized either to the housekeeping genes *Gapdh* or *Rps18*.

### Protein expression and localization

To quantify expression levels, we used standard immunoblot methods (Vazquez-Chona and Geisert, 1999). Proteins were extracted from tissue by homogenization in 2% sodium dodecyl sulfate (SDS) in 50 nM TRIS buffer (pH = 7.5). Equal quantities of proteins were separated on 4–15% SDS-polyacrylamide gel electrophoresis (SDS-PAGE) and transferred to nitrocellulose membranes. Primary antibodies included hamster anti-CD81 (Eat2; Becton Dichinson, San Jose, CA), rabbit anti-GFAP (Lipshaw), rabbit-anti ID2 (Santa Cruz Biotechnology, Santa Cruz, CA), rabbit antiezrin (Santa Cruz Biotechnology), rabbit anti-STAT3 (Santa Cruz Biotechnology), and rabbit anti-PCNA (Santa Cruz Biotechnology). Primary antibodies were detected with per-oxidase-labeled secondary anti-rabbit, anti-mouse, and anti-hamster antibodies (Jackson ImmunoResearch, West Grove, PA).

Indirect immunohistochemical methods were used to define the cellular localization of GFAP (Vazquez-Chona and Geisert, 1999). For the purpose of immunohistochemistry, deeply anesthetized animals were perfused through the heart with saline and 4% paraformaldehyde in 0.1 M phosphate buffer. Eyes were removed and fixed in 4% paraformaldehyde, stored in 30% sucrose, and sectioned to obtain 16-μm frozen sections. To retrieve cross-linked cytoplasmic antigens, we treated retinal sections with a solution of 1 mmol/L ethylene glycolbis (2-aminoethylether)-N,N,N′,N′— tetraacetic acid (EGTA, pH 8.4; Sigma-Aldrich, St. Louis, MO) and 0.3 M sucrose for 20 min at 80 °C. Sections and cells were washed in borate-buffered saline (BBS, pH 8.0; Sigma-Aldrich) and blocked in 4% bovine serum albumin (BSA; Sigma-Aldrich) and 0.1% Triton X-100 (Sigma-Aldrich). Rabbit polyclonal anti-GFAP (1:100 dilution; Lipshaw) and FITC donkey anti-rabbit IgG (1:200 dilution; Jackson ImmunoResearch, West Grove, CA) were dissolved in BBS, 0.4% BSA, and 0.1% Triton X-100. DNA was stained with 4,6-diamino-2-phenylindole (DAPI; Sigma-Aldrich), Sytox Orange (Invitrogen), and TOPRO-3 (Invitrogen). For fluorescence microscopy, sections were examined with a confocal laser-scanning microscope (MRC-1024; Bio-Rad, Hercules, CA). No immunoreactivity was observed in normal or injured retina when primary antibodies were omitted.

## Supplementary Materials

Figure S1 Expression patterns for chromosome 12 network genes during retinal development and retinal healing. A survey of temporal expression during retinal development (Dorrell et al. 2004) and wound healing (Vazquez-Chona et al. 2004) is helpful in establishing flows of action within the network. For example, the genes involved in regulating proliferation and migration in the postnatal retina (postnatal days 0 through 4) include *Id2, Zfp361l, Pax6, Tcf4, Ssb1, Nfib, Casp3,* and *Rala*; whereas genes involved in Muller glia differentiation (postnatal days 8 through 14) include the glial genes *Stat3, Gfap, Ets1*, and *Hes3*. The pattern of expression in the injured retina revealed that most of the chromosome 12 network genes are differentially expressed during the early acute and delayed subacute phases of wound healing. Network genes underlying the acute phase include the transcription regulators *Id2, Zfp36l1, Fos, Egr1, Nr4a1, Junb* and *Stat3*. Network genes underlying the subacute response include transcription regulators *Pax6, Neurod1, Crem, Nfib, Rala, Tcf4, Pou2f1, Hes3*, and *Ssb.* A select number of network genes are also differentially expressed during the chronic response. The chronic response is characterized by tissue remodeling including retinal rewiring and the formation of ectopic glial scar tissue. Chronic genes in the network include the glial markers *Stat3, Gfap, Cd81* and *Cp*.

Figure S2 *Id2* and *Lpin1* are differentially expressed in response to genetic differences and trauma. **A:** Gene expression pattern for *Id2* across forebrains from BXD recombinant mouse strains. *Id2* expression is highly heritable (F_34,99_ = 5.2, p < 10^−9^). High *Id2* expression levels correlated with the C57BL/6 allele of the chromosome 12 locus (at 10–30 Mb); whereas, low *Id2* expression levels correlated with the DBA/2J allele. Similar results were also obtained for Lpin1 (F_34,99_ = 5.1, p < 10^−9^; data not shown). Data obtained from GeneNetwork (UTHSC Brain mRNA U74Av2 HWT1PM, December 2003) (GeneNetwork). **B:** Transcript levels for *Id2* and *Lpin1* in the retina of BXD RI strains were measured with real time RT-PC. For a high *Id2/Lpin1* expresser, we used the BXD38 strain. For the low *Id2/Lpin1* expresser, we used the DBA/2J mouse strain. We also confirmed the protein levels for ID2 in selected BXD RI mouse strains. Protein levels were not measured for Lpin1. **C:** Transcript levels for *Id2* and *Lpin1* after retinal trauma. Transcripts were measured in DBA/2J retina 4 hours after a mechanical tear or vitreal injection of kanaic acid. **D:** Protein levels from injured retina also confirmed the upregulation of ID2. The upregulation of ID2 in injured retina is consistent with the upregulation of classic wound healing factors such as the acute phase factor STAT3, the reactive gliosis marker GFAP, and the proliferative maker PCNA. Ezrin (villin 2) is a house-keeping gene product. For each protein and RNA sample, retinas from two normal or injured animals were pooled (n = 4 retinas per sample).

Figure S3 *Id2* silencing decreases the mitotic activity and healing response of cultured cerebellar astrocytes. We investigated the function of ID2 in glial cells by silencing the *Id2* transcript in a mouse astrocyte cell line, the C8D1A astrocytes. C8D1A astrocytes efficiently uptake short inhibitor RNAs (siRNAs) and transport them near the nucleus (A, red channel). We designed three siRNAs to target the *Id2* mRNA (B; NM_010496, at 568, 898, and 1131) and compared their silencing efficiency against a scrambled siRNA. Off-target silencing was determined by measuring the transcript and protein changes of the reactive gliosis molecule, CD81. After a 24 h transfection, the siRNAs successfully reduced *Id2* transcript to levels below 30%. The siRNA with the best silencing efficiency was used for all experiments (93% silencing, p < 0.001; B). This siRNA targets the 3′ untranslated region of *Id2* at about 568 nucleotides from the starting site. *Id2* siRNAs produced minimal off-target effects, as shown by the non-significant changes in *Cd81* transcript levels (B). Reductions in *Id2* expression also resulted in a 33% decrease in ID2 protein levels to (*p* < 0.02, C). Protein change for off-target genes was minimal. *CD81* and ezrin protein levels were virtually unchanged (C). Therefore, our RNA interference technique successfully decreased the expression of *Id2* with minimal off-target effects. To determine functional changes due to lower ID2 levels, we used a cell culture model of wound healing induced by the loss of contact inhibition. In this model, astrocytes at the leading edge upregulate GFAP, migrate, and proliferate to repair the wound. Twenty-four hours after transfection, we induced the loss of contact inhibition by scraping cells off at the center of the monolayer. We followed morphological changes of the reactive astrocytes entering the wound using time-lapse photography. At 24 and 48 h there was a significant decrease in the rate of woundhealing closure (D). For example, migration distance of the *Id2*-siRNA treated cells was 54.8% (*p* < 10^−6^) of control-siRNA treated cells at 24 h after scratch (D). To determine if the decrease in wound healing was due to changes in cell cycle dynamics, we measured changes in mitotic rate using DNA cell cycle analysis. DNA content measurements with flow cytometry revealed that there was a 32.5% decrease in the number of cells in the S-phase (from 32.6 to 24.7%). Together these data suggest that ID2 controls the progression of astrocytes through the cell cycle. For detailed methods on cell culture and RNA interference techniques please see Vazquez (2006).

Table S1 Wound-healing genes modulated by chromosome 12 locus.

**Table:** 

		Fold Change			Cellular Expression			
Symbol	U34 Probe Set ID	Early	Delayed	Chronic	Muller	Astrocyte	Neuron	U74 Probe Set ID
*Acrv1*	rc_AI639153_at	(1.2)	(1.6)	(2.2)	+	−	+	92897_at
*bcl2*	L14680_g_at	(1.4)	(1.4)	(1.2)	+	+	+	98868_at
*casp3*	U49930_at	1.8	4.6	1.8	−	++	++	98436_s_at
*Ccl3 (Scya3)*	U22414_at	3.4	(1.7)	(1.8)	++	+	++	102424_at
*Cd81*	rc_AI103957_g_at	1.0	1.8	1.8	+++	++	+++	101495_at
*Colla1*	U75405UTR#1_f_at	1.5	12.5	37.8	+	+++	+++	103709_at
*Cp*	L33869_at	2.0	10.1	9.4	+++	+	++	92851_at
*Crem*	S66024_g_at	2.7	1.7	1.5	++	+	+	160526_s_at
*Dusp1 (Ptpn16)*	U02553cds_s_at	4.2	(1.7)	(2.1)	++	+++	++	104598_at
*Egr1*	rc_AI176662_s_at	3.4	(2.9)	(3.4)	++	+	++	161802_i_at
*Ets1*	L20681_at	(1.8)	(1.7)	(2.2)	+	+	+	94720_at
*Fos*	X06769cds_g_at	2.6	(1.1)	(1.1)	+++	+++	+++	160901_at
*Gbl*	AF051155_g_at	(2.8)	1.1	1.9	++	+	−	161602_at
*Gfap*	AF028784mRNA#1_s_at	1.3	10.4	19.1	+++	++	+	94143_at
*Ggt1*	M57672mRNA#2_s_at	(1.2)	(1.5)	(2.1)	+	+	+	100085_at
*Hes3*	D13418_at	(1.5)	(2.0)	(2.2)	+	+	+	97156_at
*Hk2*	D26393exon_s_at	(1.4)	(3.3)	(1.5)	++	+	−	161313_at
*Id2*	rc_AI230256_at	6.3	3.2	2.2	++	+++	+++	93013_at
*Itgb1*	rc_AI177366_at	2.1	3.9	2.4	++	+++	+++	100123_f_at
*Junb*	X54686cds_at	1.5	(2.3)	(1.2)	+++	+	+	102362_i_at
*Lamc1*	X94551_at	(1.2)	(1.4)	(1.7)	+	+	−	102404_at
*Lamp2*	D90211_s_at	1.8	3.3	1.9	++	++	+++	101590_at
Lpin1 *	n/a *	1.5	1.5	n/a	n/a	−	+	98892_at
*Lyz*	rc_AA892775_at	4.8	37.5	31.3	+	−	+	100611_at
*Map2k2*	rc_AA963674_at	(1.1)	(1.5)	(1.3)	+++	++	++	92543_at
*Neurod1*	D82074_g_at	1.7	2.4	1.3	+	++	+	92717_at
*Neurod2*	D82868_at	1.3	(2.5)	1.0	+	++	++	98808_at
*Nfib*	rc_AI176488_at	(2.1)	(1.2)	(1.2)	++	+++	+++	99440_at
*Nr4a1*	U17254_at	2.7	(1.4)	(1.3)	++	++	++	102371_at
*Pax6*	S74393_s_at	1.7	2.4	1.4	+	++	++	92271_at
*Pou2f1*	U17013_at	(1.3)	(2.1)	(1.0)	+	+	+	102893_at
*Ptafr*	U04740_at	1.2	(1.3)	(1.6)	+	++	+	94158_f_at
*Rab14*	M83680_at	(2.4)	(2.6)	(1.5)	+	+++	+++	97301_at
*Rala*	L19698_at	(1.7)	(2.2)	(1.5)	+	+++	++	94998_at
*Robo1*	rc_AA860017_at	(1.5)	(1.4)	1.3	−	+	+	103675_at
*Rpo1*	rc_AA799724_at	(2.1)	(1.9)	(2.2)	−	++	++	161347_r_at
*Sdc1*	S61865_s_at	(56.5)	(5.0)	(7.2)	+	++	+	161370_f_at
*Ssb*	S59893_f_at	1.1	1.0	(1.6)	+	++	++	92579_at
*Sox11*	AJ004858_at	(2.0)	(2.5)	(2.1)	−	++	+++	101631_at
*Stat3*	X91810_at	5.2	3.7	2.1	+	++	++	99099_at
*Tcf4*	U09228_at	(1.6)	(1.5)	(1.2)	++	+++	+++	160483_at
*Trib3*	rc_H31287_g_at	(1.2)	(2.7)	1.1	+	+	+	161067_at
*Vdac1*	AF048828_g_at	1.6	(1.1)	1.7	++	++	++	98139_at
*Zfp36l1*	rc_AI136891_at	2.5	1.5	1.7	+++	++	+++	93324_at

* Probe set for Lpin1 is not available in Affy U34 chip. Post meta-analysis experiments confirmed the role of Lpinl as a wound healing gene. Fold Change Cellular Expression BXD Phenotype identifiers included 10652,10655,10434-5,10437-8,10504,10338, 10340-1, and 10378. We also used the following Affymetrix probe sets for eQTL analysis of cerebellum and striatum: for Id2 were U74v2 93103_at, M430 1435176_a_at_A, and U34A rc_AI230256_at; for Lpin1 U74v2 98892_at, and M430 1426516_a_at_A; for Sox11 U74v2 101631_at and M430 1431225_at_B, and M430 1453002_at_B; and forAL024210 M430 1438758_at_Aand M430 1449076_x_at_A.

Table S2 Gene expression correlations.

**Table:** 

Symbol	Pax6	Map2k2	Ssb	Neurod1	Cp	Acrv1	Id2	Zfp36l1	Gfap	Ptafr	Ets1	Rala	Hes3	Rab14	Vdac1	Casp3	Neurod2	Bcl2	Lpin1	Stat3	Nfib	Ggt1	Itgb1	Itgb1	Lyzs	Cd81	Lamp2	Sox11	Junb	Nr4a1	Lamc1	Ccl3	Pou2f1	Rala	Robo1	Col1a1	Dusp1	Tcf4	Crem	Nfib	Fos
**Pax6**	1.00	(0.87)	0.70	0.78	0.69	0.80	0.57	0.67	(0.74)	(0.72)	(0.87)	0.69	(0.73)	0.57	(0.71)	0.83	0.64	0.58	0.68	(0.61)	0.62	(0.87)	0.44	0.65	(0.82)	0.32	0.78	0.32	0.37	0.54	(0.80)	(0.69)	0.37	0.71	0.83	0.74	0.43	0.40	0.63	0.62	0.59
**Map2k2**	(0.93)	1.00	(0.76)	(0.76)	(0.63)	0.73	(0.56)	(0.62)	0.62	0.75	0.84	(0.79)	0.76	(0.68)	0.69	(0.84)	(0.58)	(0.56)	(0.65)	0.62	(0.69)	0.90	(0.53)	(0.71)	0.79	(0.36)	(0.80)	(0.33)	(0.26)	(0.48)	0.87	0.80	(0.41)	(0.81)	(0.75)	(0.73)	(0.37)	(0.49)	(0.74)	(0.64)	(0.54)
**Ssb**	0.68	(0.71)	1.00	0.85	0.70	(0.77)	0.61	0.74	(0.45)	(0.69)	0.75	0.92	(0.72)	0.84	(0.56)	0.84	0.66	0.67	0.63	(0.62)	0.83	(0.76)	0.81	0.79	(0.64)	0.09	0.92	0.64	0.20	0.61	(0.83)	(0.68)	0.61	0.88	0.80	0.67	0.44	0.76	0.90	0.85	0.67
**Neurod1**	0.83	(0.79)	0.87	1.00	0.85	(0.87)	0.60	0.74	(0.55)	(0.78)	(0.81)	0.87	(0.74)	0.70	(0.62)	0.85	0.74	0.63	0.72	(0.69)	0.83	(0.83)	0.71	0.90	(0.84)	0.20	0.86	0.53	0.24	0.53	(0.91)	(0.76)	0.57	0.82	0.86	0.83	0.39	0.63	0.78	0.81	0.59
**Cp**	0.77	(0.70)	0.67	0.79	1.00	(0.51)	0.59	0.45	(0.59)	(0.53)	(0.64)	(0.66)	(0.41)	0.54	(0.62)	0.69	0.60	0.58	0.61	(0.46)	0.72	(0.72)	0.56	0.71	(0.76)	0.15	0.64	0.53	0.34	0.38	(0.63)	(0.63)	0.72	0.65	0.69	0.78	0.23	0.74	0.61	0.76	0.37
**Acrv1**	(0.83)	0.78	(0.76)	(0.88)	(0.59)	1.00	(0.51)	(0.82)	0.49	0.70	0.82	(0.80)	0.82	(0.69)	0.53	(0.81)	(0.77)	(0.50)	(0.65)	0.64	(0.66)	0.76	(0.62)	(0.76)	0.73	(0.20)	(0.87)	(0.44)	(0.04)	(0.51)	0.89	0.60	(0.37)	(0.77)	(0.85)	(0.74)	(0.34)	(0.37)	(0.70)	(0.62)	(0.58)
**Id2**	0.57	(0.55)	0.69	0.65	0.55	(0.54)	1.00	0.59	(0.52)	(0.54)	(0.59)	0.56	(0.56)	(0.62)	(0.55)	0.63	0.41	0.61	0.86	(0.58)	0.49	(0.63)	0.48	0.48	(0.55)	0.27	0.56	0.68	0.40	0.68	(0.62)	(0.60)	0.56	0.50	0.69	0.57	0.61	0.46	0.62	0.56	0.62
**Zfp36l1**	0.64	(0.63)	0.85	0.81	0.47	(0.78)	0.66	1.00	(0.39)	(0.66)	(0.73)	0.71	(0.86)	0.79	(0.60)	0.74	0.63	0.55	0.67	(0.59)	0.56	(0.69)	0.70	0.60	(0.58)	0.33	0.80	0.56	0.23	0.74	(0.74)	(0.56)	0.47	0.63	0.83	0.53	0.61	0.45	0.68	0.63	0.78
**Gfap**	(0.76)	0.65	(0.42)	(0.55)	(0.62)	0.52	(0.52)	(0.33)	1.00	0.56	0.64	(0.35)	0.45	(0.32)	0.74	(0.63)	(0.23)	(0.58)	(0.57)	0.37	(0.45)	0.61	(0.26)	(0.44)	0.68	(0.44)	(0.51)	(0.20)	(0.47)	(0.37)	0.54	0.51	(0.36)	(0.36)	(0.60)	(0.57)	(0.32)	(0.32)	(0.48)	(0.48)	(0.35)
**Ptafr**	(0.85)	0.84	(0.63)	(0.70)	(0.60)	0.66	(0.48)	(0.60)	0.63	1.00	0.74	(0.75)	0.79	(0.66)	0.71	(0.74)	(0.42)	(0.71)	(0.66)	0.49	(0.77)	0.82	(0.65)	(0.75)	0.80	(0.47)	(0.78)	(0.27)	(0.39)	(0.55)	0.83	0.79	(0.47)	(0.71)	(0.72)	(0.61)	(0.51)	(0.52)	(0.69)	(0.75)	(0.61)
**Ets1**	(0.92)	0.86	(0.73)	(0.83)	(0.66)	0.85	(0.60)	(0.76)	0.68	0.80	1.00	(0.76)	0.83	(0.68)	0.68	(0.91)	(0.66)	(0.61)	(0.66)	0.68	(0.61)	0.87	(0.56)	(0.66)	0.78	(0.38)	(0.86)	(0.48)	(0.26)	(0.64)	0.84	0.72	(0.40)	(0.70)	(0.77)	(0.72)	(0.48)	(0.44)	(0.76)	(0.65)	(0.69)
**Rala**	0.74	(0.78)	0.93	0.89	0.67	(0.81)	0.64	0.82	(0.37)	(0.68)	(0.78)	1.00	(0.75)	0.87	(0.56)	0.81	0.71	0.57	0.62	(0.67)	0.85	(0.80)	0.83	0.88	(0.68)	0.17	0.89	0.58	0.12	0.50	(0.88)	(0.77)	0.54	0.92	0.76	0.70	0.37	0.69	0.87	0.81	0.58
**Hes3**	(0.74)	0.77	(0.74)	(0.74)	(0.43)	0.78	0.60	(0.83)	0.47	0.74	0.85	(0.77)	1.00	(0.77)	0.63	(0.79)	(0.56)	(0.56)	(0.63)	0.66	(0.60)	0.83	(0.62)	(0.62)	0.68	(0.46)	(0.82)	(0.47)	(0.19)	(0.69)	0.83	0.67	(0.38)	(0.70)	(0.74)	(0.51)	(0.58)	(0.40)	(0.74)	(0.62)	(0.74)
**Rab14**	0.66	(0.74)	0.83	0.74	0.54	(0.71)	0.63	0.80	(0.37)	(0.69)	(0.73)	0.87	(0.80)	1.00	(0.59)	0.73	0.56	0.54	0.59	(0.58)	0.67	(0.70)	0.86	0.67	(0.51)	0.31	0.82	0.71	0.19	0.67	(0.74)	(0.69)	0.58	0.79	0.67	0.50	0.47	0.67	0.83	0.73	0.68
**Vdac1**	(0.85)	0.82	(0.58)	(0.67)	(0.62)	0.63	(0.55)	(0.61)	0.75	0.90	0.82	(0.61)	0.72	(0.69)	1.00	(0.67)	(0.32)	(0.57)	(0.60)	0.38	(0.60)	0.76	(0.55)	(0.60)	0.69	(0.59)	(0.65)	(0.36)	(0.51)	(0.58)	0.66	0.74	(0.36)	(0.48)	(0.68)	(0.50)	(0.53)	(0.56)	(0.63)	(0.65)	(0.52)
**Casp3**	0.84	(0.80)	0.85	0.87	0.70	(0.82)	0.65	0.80	(0.60)	(0.64)	(0.89)	0.84	(0.82)	0.76	(0.67)	1.00	0.60	0.59	0.69	(0.64)	0.69	(0.86)	0.68	0.72	(0.77)	0.28	0.89	0.53	0.24	0.63	(0.83)	(0.70)	0.47	0.81	0.82	0.71	0.41	0.58	0.84	0.73	0.70
**Neurod2**	0.62	(0.56)	0.65	0.78	0.58	(0.80)	0.44	0.69	(0.26)	(0.39)	(0.69)	0.74	(0.54)	0.53	(0.41)	0.67	1.00	0.37	0.52	(0.50)	0.60	(0.59)	0.48	0.67	(0.55)	(0.12)	0.71	0.43	(0.11)	0.35	(0.68)	(0.45)	0.45	0.70	0.68	0.68	0.12	0.39	0.59	0.58	0.37
**Bcl2**	0.66	(0.60)	0.78	0.75	0.60	(0.61)	0.63	0.75	(0.56)	(0.73)	(0.72)	0.75	(0.63)	0.65	(0.67)	0.68	0.57	1.00	0.62	(0.27)	0.72	(0.58)	0.57	0.56	(0.60)	0.16	0.68	0.39	0.37	0.51	(0.61)	(0.52)	0.63	0.54	0.67	0.48	0.47	0.64	0.67	0.78	0.56
**Lpin1**	0.69	(0.66)	0.70	0.76	0.59	(0.69)	0.87	0.72	(0.53)	(0.63)	(0.68)	0.68	(0.66)	0.60	(0.64)	0.71	0.55	0.69	1.00	(0.60)	0.61	(0.70)	0.55	0.66	(0.72)	0.40	0.63	0.60	0.49	0.70	(0.72)	(0.65)	0.57	0.56	0.84	0.65	0.62	0.46	0.58	0.62	0.66
**Stat3**	(0.65)	0.65	(0.59)	(0.59)	(0.38)	0.64	(0.52)	(0.55)	0.33	0.57	0.68	(0.65)	0.68	(0.59)	0.47	(0.59)	(0.44)	(0.33)	(0.53)	1.00	(0.40)	0.67	(0.39)	(0.56)	0.52	(0.38)	(0.55)	(0.53)	(0.28)	(0.56)	0.69	0.64	(0.32)	(0.56)	(0.57)	(0.59)	(0.55)	(0.22)	(0.52)	(0.36)	(0.60)
**Nfib**	0.69	(0.69)	0.84	0.85	0.79	(0.66)	0.54	0.65	(0.48)	(0.69)	(0.65)	0.86	(0.57)	0.69	(0.63)	0.72	0.63	0.79	0.64	(0.36)	1.00	(0.74)	0.78	0.87	(0.74)	0.07	0.83	0.42	0.21	0.40	(0.79)	(0.67)	0.64	0.79	0.74	0.65	0.27	0.80	0.77	0.95	0.43
**Ggt1**	(0.94)	0.95	(0.70)	(0.82)	(0.73)	0.77	(0.57)	(0.66)	0.65	0.90	0.89	(0.76)	0.82	(0.73)	0.88	(0.80)	(0.56)	(0.63)	(0.69)	0.68	(0.71)	1.00	(0.61)	(0.75)	0.85	(0.45)	(0.83)	(0.47)	(0.37)	(0.61)	0.90	0.79	(0.44)	(0.75)	(0.81)	(0.73)	(0.49)	(0.54)	(0.76)	(0.73)	(0.65)
**Itgb1**	0.48	(0.51)	0.86	0.76	0.49	(0.63)	0.58	0.83	(0.21)	(0.52)	(0.59)	0.86	(0.65)	0.82	(0.49)	0.72	0.56	0.74	0.62	(0.38)	0.77	(0.54)	1.00	0.77	(0.52)	0.23	0.80	0.66	0.15	0.52	(0.65)	(0.57)	0.53	0.73	0.68	0.44	0.32	0.78	0.79	0.82	0.56
**Itgb1**	0.84	(0.83)	0.82	0.94	0.78	(0.83)	0.54	0.73	(0.52)	(0.78)	(0.80)	0.89	(0.71)	0.76	(0.72)	0.82	0.70	0.72	0.71	(0.57)	0.87	(0.86)	0.77	1.00	(0.80)	0.25	0.78	0.46	0.22	0.38	(0.84)	(0.73)	0.55	0.83	0.77	0.74	0.27	0.66	0.74	0.78	0.45
**Lyzs**	(0.91)	0.85	(0.65)	(0.87)	(0.84)	0.76	(0.51)	(0.60)	0.72	0.83	0.81	(0.71)	0.66	(0.59)	0.80	(0.77)	(0.57)	(0.67)	(0.70)	0.49	(0.78)	0.90	(0.52)	(0.90)	1.00	(0.43)	(0.70)	(0.32)	(0.36)	(0.43)	0.81	0.73	(0.54)	(0.69)	(0.78)	(0.82)	(0.33)	(0.52)	(0.59)	(0.69)	(0.45)
**Cd81**	0.62	(0.62)	0.28	0.39	0.34	(0.38)	0.33	0.37	(0.55)	(0.81)	(0.59)	0.32	(0.57)	0.45	(0.80)	0.38	0.06	0.43	0.48	(0.48)	0.30	(0.72)	0.26	0.51	(0.64)	1.00	0.16	0.17	0.53	0.40	(0.30)	(0.49)	0.07	0.09	0.25	0.16	0.49	0.06	0.15	0.06	0.37
**Lamp2**	0.73	(0.73)	0.93	0.89	0.64	(0.82)	0.64	0.88	(0.43)	(0.64)	(0.82)	0.93	(0.80)	0.82	(0.62)	0.90	0.75	0.80	0.68	(0.51)	0.83	(0.73)	0.87	0.84	(0.69)	0.27	1.00	0.53	0.14	0.61	(0.88)	(0.68)	0.53	0.84	0.86	0.66	0.40	0.66	0.90	0.86	0.67
**Sox11**	0.35	(0.33)	0.67	0.61	0.44	(0.50)	0.71	0.66	(0.22)	(0.26)	(0.52)	0.65	(0.49)	0.64	(0.36)	0.58	0.59	0.56	0.63	(0.45)	0.51	(0.38)	0.73	0.53	(0.33)	0.18	0.63	1.00	0.18	0.62	(0.48)	(0.40)	0.52	0.47	0.53	0.36	0.43	0.61	0.60	0.53	0.59
**Junb**	0.48	(0.40)	0.25	0.31	0.41	(0.13)	0.42	0.27	(0.50)	(0.58)	(0.39)	0.18	(0.27)	0.28	(0.61)	0.29	(0.02)	0.42	0.49	(0.28)	0.30	(0.52)	0.17	0.31	(0.49)	0.67	0.19	0.17	1.00	0.63	(0.22)	(0.42)	0.21	0.03	0.35	0.25	0.74	0.29	0.16	0.29	0.57
**Nr4a1**	0.55	(0.55)	0.68	0.59	0.36	(0.51)	0.69	0.80	(0.34)	(0.61)	(0.65)	0.60	(0.74)	0.69	(0.60)	0.66	0.36	0.64	0.71	(0.53)	0.48	(0.63)	0.63	0.51	(0.49)	0.51	0.67	0.53	0.64	1.00	(0.55)	(0.52)	0.35	0.38	0.64	0.33	0.87	0.44	0.60	0.54	0.93
**Lamc1**	(0.93)	0.93	(0.73)	(0.87)	(0.73)	0.85	(0.56)	(0.69)	0.62	0.88	0.88	(0.81)	0.80	(0.75)	0.84	(0.80)	(0.63)	(0.64)	(0.72)	0.68	(0.74)	0.97	(0.57)	(0.90)	0.91	(0.64)	(0.76)	(0.39)	(0.44)	(0.59)	1.00	0.81	(0.49)	(0.81)	(0.84)	(0.81)	(0.44)	(0.54)	(0.77)	(0.75)	(0.62)
**Ccl3**	(0.86)	0.87	(0.66)	(0.75)	(0.66)	0.67	(0.58)	(0.62)	0.63	0.93	0.82	(0.71)	0.73	(0.72)	0.90	(0.68)	(0.44)	(0.69)	(0.67)	0.65	(0.66)	0.91	(0.53)	(0.80)	0.83	(0.78)	(0.65)	(0.37)	(0.60)	(0.59)	0.90	1.00	(0.51)	(0.72)	(0.66)	(0.68)	(0.52)	(0.60)	(0.65)	(0.66)	(0.52)
**Pou2f1**	0.46	(0.47)	0.65	0.63	0.70	(0.41)	0.56	0.59	(0.35)	(0.48)	(0.47)	0.61	(0.45)	0.59	(0.44)	0.56	0.44	0.67	0.55	(0.29)	0.65	(0.49)	0.58	0.61	(0.59)	0.23	0.63	0.50	0.29	0.44	(0.52)	(0.55)	1.00	0.56	0.56	0.55	0.22	0.69	0.51	0.69	0.35
**Rala**	0.67	(0.74)	0.86	0.81	0.59	(0.77)	0.53	0.73	(0.27)	(0.54)	(0.70)	0.92	(0.73)	0.77	(0.46)	0.83	0.73	0.62	0.59	(0.56)	0.75	(0.67)	0.74	0.81	(0.62)	0.17	0.88	0.54	0.02	0.49	(0.72)	(0.58)	0.56	1.00	0.72	0.66	0.21	0.65	0.82	0.74	0.49
**Robo1**	0.88	(0.83)	0.85	0.92	0.72	(0.87)	0.70	0.87	(0.61)	(0.79)	(0.86)	0.85	(0.80)	0.78	(0.80)	0.87	0.69	0.80	0.84	(0.57)	0.79	(0.86)	0.75	0.88	(0.86)	0.53	0.88	0.55	0.45	0.72	(0.88)	(0.80)	0.63	0.75	1.00	0.72	0.51	0.56	0.72	0.77	0.68
**Col1a1**	0.86	(0.82)	0.62	0.81	0.83	(0.78)	0.54	0.52	(0.60)	(0.64)	(0.75)	0.68	(0.57)	0.54	(0.62)	0.75	0.64	0.47	0.62	(0.55)	0.64	(0.81)	0.40	0.80	(0.86)	0.37	0.64	0.31	0.28	0.36	(0.86)	(0.71)	0.53	0.64	0.75	1.00	0.24	0.46	0.55	0.61	0.35
**Dusp1**	0.53	(0.50)	0.53	0.46	0.31	(0.38)	0.66	0.65	(0.38)	(0.68)	(0.57)	0.46	(0.63)	0.54	(0.68)	0.46	0.22	0.60	0.66	(0.55)	0.38	(0.62)	0.44	0.41	(0.45)	0.65	0.47	0.41	0.78	0.88	(0.56)	(0.67)	0.35	0.28	0.64	0.29	1.00	0.27	0.42	0.37	0.83
**Tcf4**	0.45	(0.48)	0.79	0.67	0.67	(0.40)	0.54	0.57	(0.37)	(0.49)	(0.46)	0.71	(0.40)	0.66	(0.52)	0.58	0.43	0.73	0.51	(0.18)	0.86	(0.48)	0.78	0.66	(0.53)	0.18	0.70	0.62	0.33	0.46	(0.49)	(0.52)	0.63	0.59	0.61	0.39	0.37	1.00	0.70	0.87	0.43
**Crem**	0.57	(0.62)	0.88	0.76	0.50	(0.65)	0.65	0.80	(0.36)	(0.60)	(0.70)	0.85	(0.76)	0.82	(0.58)	0.79	0.61	0.80	0.61	(0.43)	0.74	(0.63)	0.83	0.72	(0.53)	0.26	0.92	0.65	0.18	0.71	(0.65)	(0.59)	0.58	0.81	0.75	0.46	0.52	0.70	1.00	0.82	0.66
**Nfib**	0.66	(0.64)	0.87	0.84	0.76	(0.65)	0.60	0.71	(0.47)	(0.65)	(0.67)	0.85	(0.59)	0.74	(0.64)	0.74	0.66	0.83	0.64	(0.31)	0.97	(0.67)	0.82	0.82	(0.71)	0.25	0.87	0.60	0.32	0.55	(0.70)	(0.64)	0.67	0.74	0.79	0.59	0.44	0.91	0.81	1.00	0.55
**Fos**	0.54	(0.55)	0.76	0.64	0.34	(0.55)	0.70	0.85	(0.29)	(0.58)	(0.64)	0.69	(0.78)	0.72	(0.54)	0.71	0.42	0.69	0.71	(0.57)	0.52	(0.61)	0.71	0.56	(0.46)	0.41	0.74	0.57	0.53	0.95	(0.59)	(0.57)	0.46	0.61	0.75	0.34	0.84	0.50	0.78	0.59	1.00
**Cd81**	(0.80)	0.76	(0.75)	(0.86)	(0.69)	0.86	(0.45)	(0.69)	0.54	0.71	0.81	(0.78)	0.71	(0.70)	0.64	(0.80)	(0.70)	(0.68)	(0.60)	0.48	(0.73)	0.77	(0.64)	(0.86)	0.80	(0.40)	(0.82)	(0.39)	(0.25)	(0.50)	0.84	0.71	(0.56)	(0.74)	(0.83)	(0.79)	(0.36)	(0.50)	(0.69)	(0.72)	(0.52)
**Trib3**	(0.87)	0.83	(0.70)	(0.74)	(0.57)	0.77	(0.56)	(0.75)	0.58	0.83	0.88	0.73	0.81	(0.73)	0.83	(0.74)	(0.60)	(0.67)	(0.65)	0.72	(0.61)	0.88	(0.51)	(0.72)	0.73	(0.64)	(0.70)	(0.43)	(0.49)	(0.66)	0.88	0.86	(0.46)	(0.61)	(0.82)	(0.67)	(0.70)	(0.44)	(0.61)	(0.62)	(0.67)
**Hk2**	(0.84)	0.78	(0.75)	(0.90)	0.65	0.95	(0.57)	(0.74)	0.52	0.68	0.84	0.80	0.74	(0.69)	0.63	(0.82)	(0.74)	(0.60)	(0.75)	0.67	(0.67)	0.79	(0.61)	(0.86)	0.82	(0.45)	(0.79)	(0.49)	(0.24)	(0.54)	0.87	0.72	(0.47)	(0.75)	(0.88)	(0.80)	(0.41)	(0.41)	(0.62)	(0.64)	(0.56)
**Rpo1-1**	0.90	(0.89)	0.66	0.86	0.76	(0.82)	0.54	0.64	(0.60)	(0.78)	(0.84)	0.75	(0.66)	0.63	(0.78)	0.76	0.71	0.59	0.70	(0.61)	0.67	(0.89)	0.49	0.88	(0.88)	0.55	0.70	0.38	0.37	0.46	(0.94)	(0.84)	0.51	0.66	0.84	0.91	0.45	0.42	0.55	0.64	0.46
**Sdc1**	(0.91)	0.91	(0.68)	(0.74)	(0.65)	0.73	(0.57)	(0.69)	0.62	0.87	0.85	(0.73)	0.77	(0.73)	0.89	(0.75)	(0.54)	(0.62)	(0.69)	0.70	(0.64)	0.93	(0.52)	(0.78)	0.81	(0.73)	(0.68)	(0.40)	(0.55)	(0.63)	0.89	0.92	(0.47)	(0.64)	(0.85)	(0.71)	(0.67)	(0.49)	(0.55)	(0.62)	(0.61)
**Gbl**	(0.77)	0.73	(0.74)	(0.87)	(0.59)	0.93	(0.52)	(0.74)	0.42	0.61	0.79	(0.79)	0.73	(0.65)	0.55	(0.81)	(0.77)	(0.57)	(0.68)	0.60	(0.62)	0.74	(0.60)	(0.82)	0.75	(0.32)	(0.81)	(0.44)	(0.10)	(0.51)	0.83	0.64	(0.47)	(0.77)	(0.84)	(0.80)	(0.34)	(0.35)	(0.68)	(0.61)	(0.54)
**Fos**	0.66	(0.66)	0.72	0.75	0.52	(0.63)	0.60	0.73	(0.35)	(0.60)	(0.76)	0.73	(0.75)	0.63	(0.56)	0.75	0.60	0.60	0.57	(0.71)	0.54	(0.70)	0.61	0.65	(0.56)	0.37	0.74	0.56	0.33	0.66	(0.68)	(0.67)	0.46	0.66	0.71	0.57	0.63	0.45	0.70	0.59	0.73
**Egr1**	0.84	(0.80)	0.56	0.70	0.66	(0.63)	0.53	0.57	(0.59)	(0.72)	(0.78)	0.62	(0.59)	0.48	(0.71)	0.69	0.58	0.59	0.61	(0.62)	0.52	(0.77)	0.39	0.70	(0.74)	0.55	0.57	0.35	0.42	0.45	(0.74)	(0.78)	0.44	0.55	0.71	0.75	0.50	0.36	0.45	0.49	0.47
**ProbeID**	**92271_at**	**92543_at**	**92579_at**	**92717_at**	**92851_at**	**92897_at**	**93013_at**	**93324_at**	**94143_at**	**94158_f_at**	**94720_at**	**94998_at**	**97156_at**	**97301_at**	**98139_at**	**98436_S_at**	**98808_at**	**98868_at**	**98892_at**	**99099_at**	**99440_at**	**100085_at**	**100123_f_at**	**100124_r_at**	**100611_at**	**101495_at**	**101590_at**	**101631_at**	**102362_i_at**	**102371_at**	**102404_at**	**102424_at**	**102893_at**	**103067_at**	**103675_at**	**103709_at**	**104598_at**	**160483_at**	**160526_s_at**	**160859_s_at**	**160901_at**

Table S3 Oligonucleotides used for real-time RT-PCR.

**Table:** 

Targets	Accession No.	Sequence (5′→3′)
*Id2*	NM_010496	GTCCTTGCAGGCATCTGAAT
		CTTAGTTTTCCTTCCGCTTTCTT
*Lpin1*	NM_015763	CCCCATTCCTCATAGCTCAA
		CACTAGTGGCTCCTCCTTGC
*Sox11*	NM_009234	CTGGTGGATAAGGACCTGGATTC
		GATCATCTCGCTCAGCTCCG
*Fos*	NM_010234	AGAATCCGAAGGGAACGGAA
		GGTCGTTGAGAAGGGGCAG
*Stat3*	NM_21365	TGTTGGAGCAGCATCTTCAG
		CTTGGCTCTTGAGGGTTTTG
*Casp3*	NM_00981	CCTCAGAGAGACATTCATGGC
		TCGGCTTTCCAGTCAGACTC
*Crygd*	NM_007776	AGCAGTGGATGGGTTTCAG
		GTGGAATCGGTCCTGGAG
*Cd81*	NM_133655	CTGTTTGCCTGTGAGGTGG
		TCAGTGTGGTCAGTGCGTT
*Gfap*	NM_010277	AGGGACAATCTCACACAGGAC
		CTCCAGCGACTCAACCTTC
*Cryab*	NM_009964	CTCTGTGAATCTGGACGTGAA
		CACCTGTTTCCTTGGTCCAT
*Cryba4*	NM_021351	TGGCTACCGAGGTTTTCAGT
		GGACACAAGGGTAGCCAGAA
*Gapdh*	NM_001001303	TCCCACTCTTCCACCTTCGATG
		GTCCACCACCCTGTTGCTGTA
*Rps18*	XM_215328	CTCGCTCCTCTCCTACTTGG
		ACCGGGTTGGTTTTGATCT

## Figures and Tables

**Table 1:** Bioinformatic analyses and online databases.

Analysis	Database*	Website
Gene expression		
Retinal development	Retina developmental gene expression	www.scripps.edu/cb/friedlander/gene_expression
	Mouse retina SAGE library	http://bricweb.partners.org/cepko/default.asp
All tissues	Gene expression Omnibus (GEO)	www.ncbi.hlm.nih.gov/geo
Expression QTL	GeneNetwork	www.genenetwork.org
Genes within QTLs	Genome browser	http://genome.ucsc.edu
	Ensembl	www.ensembl.org/Mus_musculus
	NCBI MapViewer	www.ncbi.nlm.nih.gov/mapview
Genes and loci causing Retinal diseases	RetNet	www.sph.uth.tmc.edu/Retnet/
Loci associated with neurological phenotypes	BXD published Phenotypes data-base	www.genenetwork.org
Single nucleotide polymorphisms (SNPs)	SNP browser	www.genenetwork.org/beta/snpBrowser.py?
	Ensembl mouse SNPView	www.ensembl.org/Mus_musculus
	Entrez SNP data- bases	www.ncbi.nlm.nih.gov/SNP
Functional motifs		
Transcription factor binding sites	MOTIF	http://motif.genome.jp
Protein domains	Scansite	http://scansite.mit.edu
Cellular distribution of transcript		
Retina	Mouse retina SAGE library	http://bricweb.partners.org/cepko/default.asp
Brain	Gene expression Nervous system Atlas (GENSAT)	www.ncbi.nlm.nih.gov/gensat
Gene ontology	WEB-based GEne SeT AnaLysis Toolkit (WebGestalt)	http://bioinfo.vanderbilt.edu/webgestalt/
Mining NCBI literature for interactions	Chilibot	www.chilibot.net

* Specific versions of databases are listed here: UTHSC Brain mRNA U74Av2 HWT1PM, December 2003; GNF Hematopoietic Cells U74Av2 RMA, March 2004; HBP/Rosen Striatum M430V2 RMA, April 2005; SJUT Cerebellum mRNA M430 RMA, March 2005; BXDphenotypes, Accessed November 2005; Ensembl, v33; MOTIF, Accessed November 2005; GenomeBrowser, Mouse May 2004 assembly; RetDevExpression, accessed January 2005; WebGestalt, Accessed October 2005; Chilibot, Accessed October 2005.
