# Supplementary figures and images for: Genomic Loci Modulating the Retinal Transcriptome in Wound Healing
Source: Gene Regul Syst Bio. 2008 Feb 14;1:327–48. (PMC2759132)

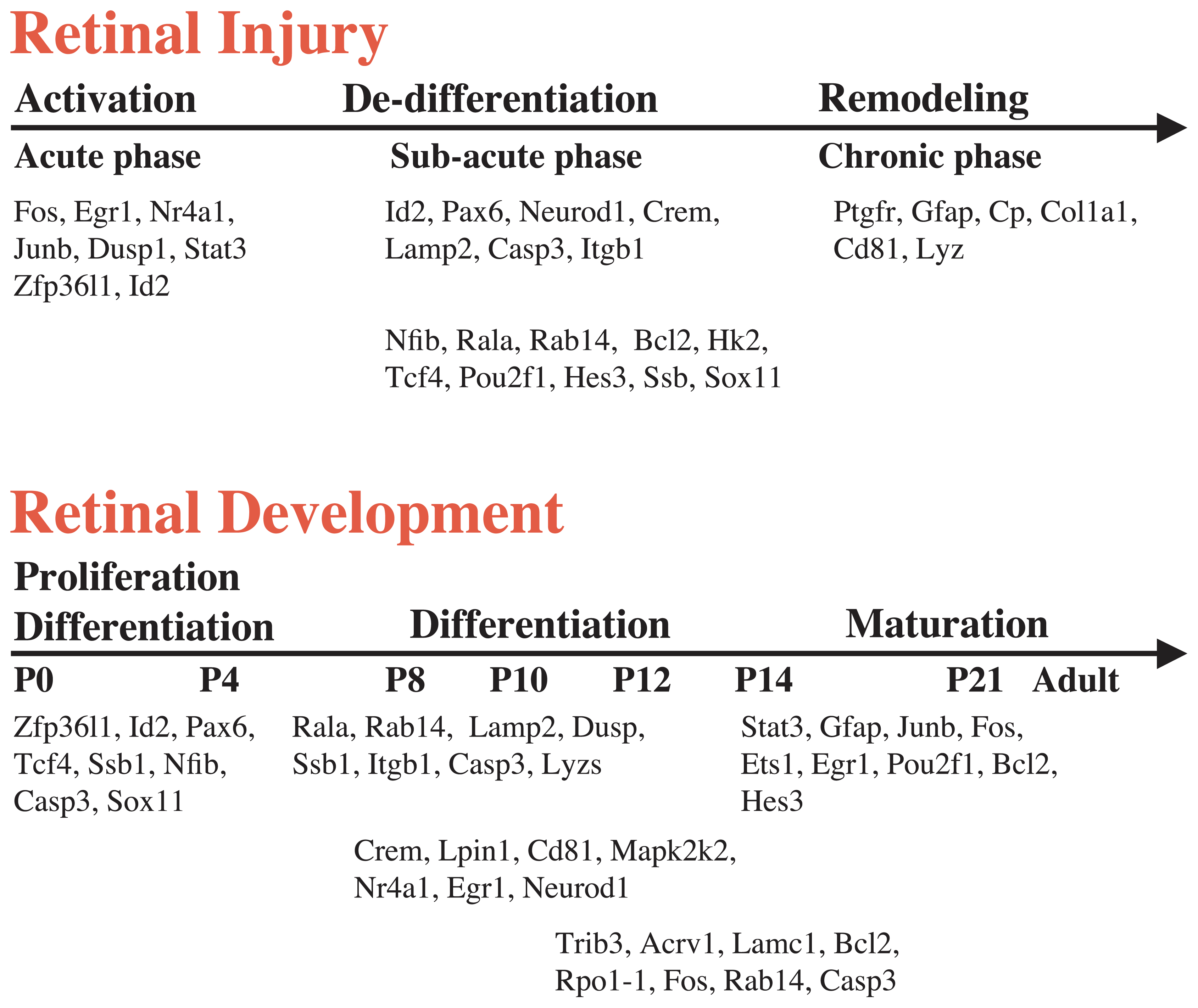

Supplement: Figure S1 — Expression patterns for chromosome 12 network genes during retinal development and retinal healing. A survey of temporal expression during retinal development (Dorrell et al. 2004) and wound healing (Vazquez-Chona et al. 2004) is helpful in establishing flows of action within the network. For example, the genes involved in regulating proliferation and migration in the postnatal retina (postnatal days 0 through 4) include Id2, Zfp361l, Pax6, Tcf4, Ssb1, Nfib, Casp3, and Rala; whereas genes involved in Muller glia differentiation (postnatal days 8 through 14) include the glial genes Stat3, Gfap, Ets1, and Hes3. The pattern of expression in the injured retina revealed that most of the chromosome 12 network genes are differentially expressed during the early acute and delayed subacute phases of wound healing. Network genes underlying the acute phase include the transcription regulators Id2, Zfp36l1, Fos, Egr1, Nr4a1, Junb and Stat3. Network genes underlying the subacute response include transcription regulators Pax6, Neurod1, Crem, Nfib, Rala, Tcf4, Pou2f1, Hes3, and Ssb. A select number of network genes are also differentially expressed during the chronic response. The chronic response is characterized by tissue remodeling including retinal rewiring and the formation of ectopic glial scar tissue. Chronic genes in the network include the glial markers Stat3, Gfap, Cd81 and Cp. [file grsb-2007-327f6.tif]

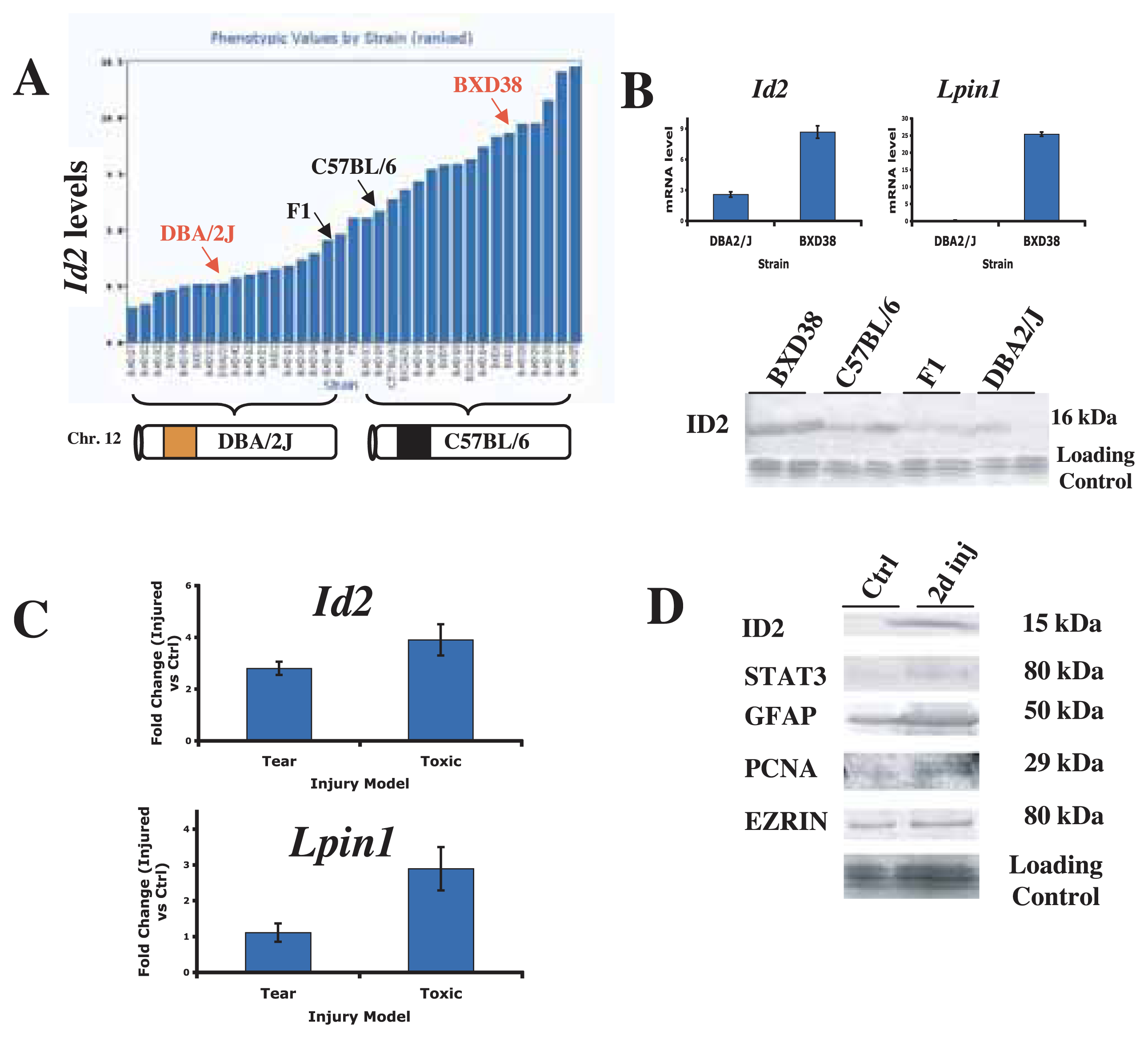

Supplement: Figure S2 — Id2 and Lpin1 are differentially expressed in response to genetic differences and trauma. A: Gene expression pattern for Id2 across forebrains from BXD recombinant mouse strains. Id2 expression is highly heritable (F34,99 = 5.2, p < 10−9). High Id2 expression levels correlated with the C57BL/6 allele of the chromosome 12 locus (at 10–30 Mb); whereas, low Id2 expression levels correlated with the DBA/2J allele. Similar results were also obtained for Lpin1 (F34,99 = 5.1, p < 10−9; data not shown). Data obtained from GeneNetwork (UTHSC Brain mRNA U74Av2 HWT1PM, December 2003) (GeneNetwork). B: Transcript levels for Id2 and Lpin1 in the retina of BXD RI strains were measured with real time RT-PC. For a high Id2/Lpin1 expresser, we used the BXD38 strain. For the low Id2/Lpin1 expresser, we used the DBA/2J mouse strain. We also confirmed the protein levels for ID2 in selected BXD RI mouse strains. Protein levels were not measured for Lpin1. C: Transcript levels for Id2 and Lpin1 after retinal trauma. Transcripts were measured in DBA/2J retina 4 hours after a mechanical tear or vitreal injection of kanaic acid. D: Protein levels from injured retina also confirmed the upregulation of ID2. The upregulation of ID2 in injured retina is consistent with the upregulation of classic wound healing factors such as the acute phase factor STAT3, the reactive gliosis marker GFAP, and the proliferative maker PCNA. Ezrin (villin 2) is a house-keeping gene product. For each protein and RNA sample, retinas from two normal or injured animals were pooled (n = 4 retinas per sample). [file grsb-2007-327f7.tif]

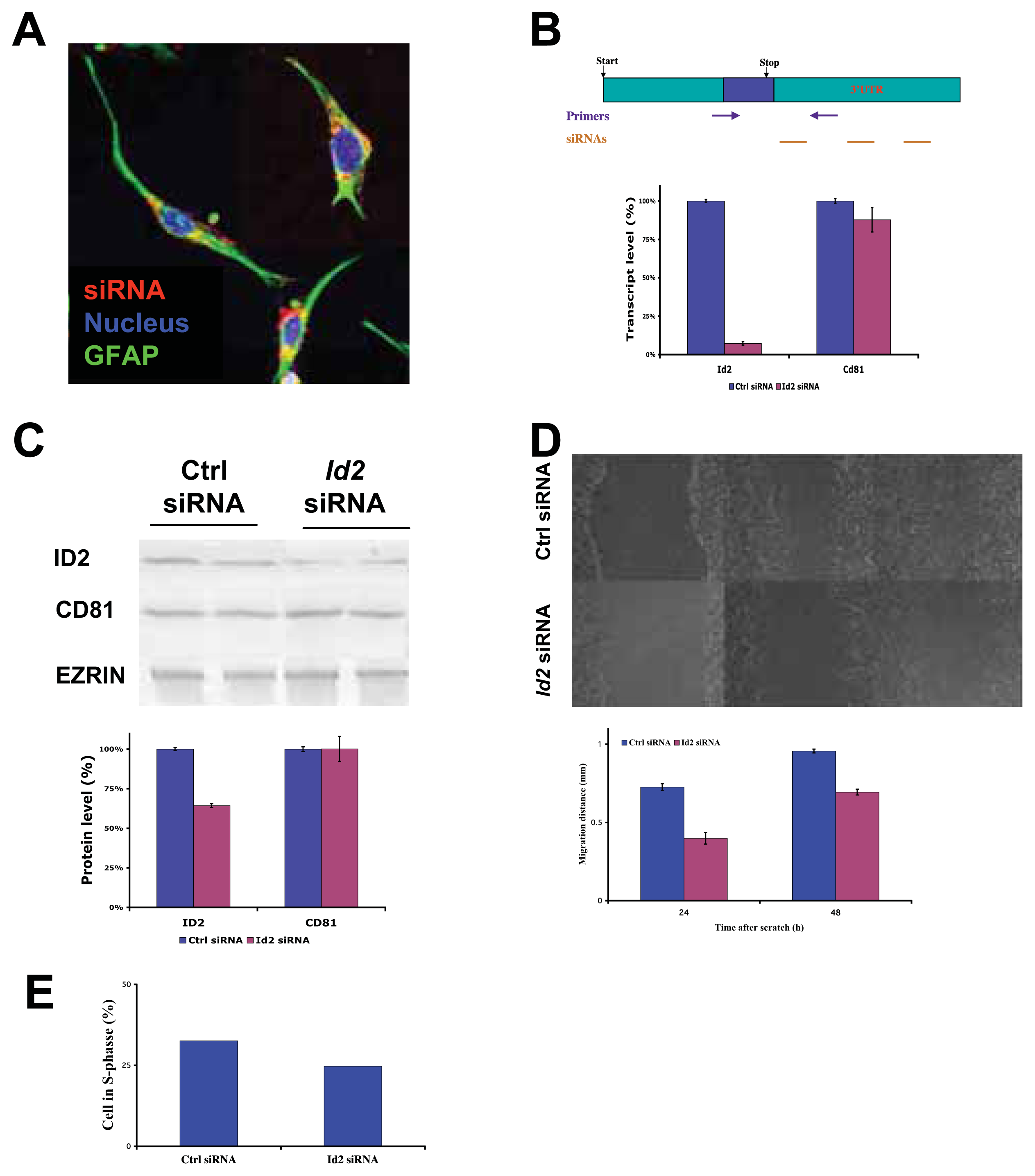

Supplement: Figure S3 — Id2 silencing decreases the mitotic activity and healing response of cultured cerebellar astrocytes. We investigated the function of ID2 in glial cells by silencing the Id2 transcript in a mouse astrocyte cell line, the C8D1A astrocytes. C8D1A astrocytes efficiently uptake short inhibitor RNAs (siRNAs) and transport them near the nucleus (A, red channel). We designed three siRNAs to target the Id2 mRNA (B; NM_010496, at 568, 898, and 1131) and compared their silencing efficiency against a scrambled siRNA. Off-target silencing was determined by measuring the transcript and protein changes of the reactive gliosis molecule, CD81. After a 24 h transfection, the siRNAs successfully reduced Id2 transcript to levels below 30%. The siRNA with the best silencing efficiency was used for all experiments (93% silencing, p < 0.001; B). This siRNA targets the 3′ untranslated region of Id2 at about 568 nucleotides from the starting site. Id2 siRNAs produced minimal off-target effects, as shown by the non-significant changes in Cd81 transcript levels (B). Reductions in Id2 expression also resulted in a 33% decrease in ID2 protein levels to (p < 0.02, C). Protein change for off-target genes was minimal. CD81 and ezrin protein levels were virtually unchanged (C). Therefore, our RNA interference technique successfully decreased the expression of Id2 with minimal off-target effects. To determine functional changes due to lower ID2 levels, we used a cell culture model of wound healing induced by the loss of contact inhibition. In this model, astrocytes at the leading edge upregulate GFAP, migrate, and proliferate to repair the wound. Twenty-four hours after transfection, we induced the loss of contact inhibition by scraping cells off at the center of the monolayer. We followed morphological changes of the reactive astrocytes entering the wound using time-lapse photography. At 24 and 48 h there was a significant decrease in the rate of woundhealing closure (D). For example, migration dis [file grsb-2007-327f8.tif]
